# Associations between an IgG3 polymorphism in the binding domain for FcRn, transplacental transfer of malaria-specific IgG3, and protection against *Plasmodium falciparum* malaria during infancy: A birth cohort study in Benin

**DOI:** 10.1371/journal.pmed.1002403

**Published:** 2017-10-09

**Authors:** Celia Dechavanne, Sebastien Dechavanne, Ibrahim Sadissou, Adjimon Gatien Lokossou, Fernanda Alvarado, Magalie Dambrun, Kabirou Moutairou, David Courtin, Gregory Nuel, Andre Garcia, Florence Migot-Nabias, Christopher L. King

**Affiliations:** 1 Center for Global Health and Diseases, Case Western Reserve University, Cleveland, Ohio, United States of America; 2 Mère et Enfant Face aux Infections Tropicales, UMR 216, Institut de Recherche pour le Développement, Paris, France; 3 Faculté de Pharmacie, Université Paris Descartes, COMUE Sorbonne Paris Cité, Paris, France; 4 Division of Clinical Immunology, School of Medicine of Ribeirão Preto, University of São Paulo, São Paulo, Brazil; 5 Laboratoire de Recherche en Biologie Appliquée, Unité de Recherche Sciences Biomédicales et Environnement, École Polytechnique d’Abomey Calavi, Université d’Abomey Calavi, Abomey Calavi, Benin; 6 Laboratoire de Biologie et Physiologie Cellulaires, Faculté des Sciences et Techniques, Université d’Abomey-Calavi, Abomey Calavi, Benin; 7 Laboratoire de Mathématiques Appliquées, UMR CNRS 8145, Université Paris Descartes, Paris, France; 8 Veterans Affairs Research Service, Cleveland, Ohio, United States of America; Mahidol-Oxford Tropical Medicine Research Unit, THAILAND

## Abstract

**Background:**

Transplacental transfer of maternal immunoglobulin G (IgG) to the fetus helps to protect against malaria and other infections in infancy. Recent studies have emphasized the important role of malaria-specific IgG3 in malaria immunity, and its transfer may reduce the risk of malaria in infancy. Human IgGs are actively transferred across the placenta by binding the neonatal Fc receptor (FcRn) expressed within the endosomes of the syncytiotrophoblastic membrane. Histidine at position 435 (H435) provides for optimal Fc–IgG binding. In contrast to other IgG subclasses, IgG3 is highly polymorphic and usually contains an arginine at position 435, which reduces its binding affinity to FcRn in vitro. The reduced binding to FcRn is associated with reduced transplacental transfer and reduced half-life of IgG3 in vivo. Some haplotypes of IgG3 have histidine at position 435. This study examines the hypotheses that the IgG3-H435 variant promotes increased transplacental transfer of malaria-specific antibodies and a prolonged IgG3 half-life in infants and that its presence correlates with protection against clinical malaria during infancy.

**Methods and findings:**

In Benin, 497 mother–infant pairs were included in a longitudinal birth cohort. Both maternal and cord serum samples were assayed for levels of IgG1 and IgG3 specific for MSP1_19_, MSP2 (both allelic families, 3D7 and FC27), MSP3, GLURP (both regions, R0 and R2), and AMA1 antigens of *Plasmodium falciparum*. Cord:maternal ratios were calculated. The maternal IgG3 gene was sequenced to identify the IgG3-H435 polymorphism. A multivariate logistic regression was used to examine the association between maternal IgG3-H435 polymorphism and transplacental transfer of IgG3, adjusting for hypergammaglobulinemia, maternal malaria, and infant malaria exposure. Twenty-four percent of Beninese women living in an area highly endemic for malaria had the IgG3-H435 allele (377 women homozygous for the IgG3-R435 allele, 117 women heterozygous for the IgG3-R/H alleles, and 3 women homozygous for the IgG3-H435 allele). Women with the IgG3-H435 allele had a 78% (95% CI 17%, 170%, *p =* 0.007) increased transplacental transfer of GLURP-R2 IgG3 compared to those without the IgG3-H435 allele. Furthermore, in infants born to mothers with the IgG3-H435 variant, a 28% longer IgG3 half-life was noted (95% CI 4%, 59%, *p =* 0.02) compared to infants born to mothers homozygous for the IgG3-R435 allele. Similar findings were observed for AMA1, MSP2-3D7, MSP3, GLURP-R0, and GLURP-R2 but not for MSP1_19_ and MSP2-FC27. Infants born to women with IgG3-H435 had a 32% lower risk of symptomatic malaria during infancy (incidence rate ratio [IRR] = 0.68 [95% CI 0.51, 0.91], *p =* 0.01) compared to infants born to mothers homozygous for IgG3-R435. We did not find a lower risk of asymptomatic malaria in infants born to women with or without IgG3-H435. Limitations of the study were the inability to determine (i) the actual amount of IgG3-H435 relative to IgG-R435 in serum samples and (ii) the proportion of malaria-specific IgG produced by infants versus acquired from their mothers.

**Conclusions:**

An arginine-to-histidine replacement at residue 435 in the binding domain of IgG3 to FcRn increases the transplacental transfer and half-life of malaria-specific IgG3 in young infants and is associated with reduced risk of clinical malaria during infancy. The IgG3-H435 allele may be under positive selection, given its relatively high frequency in malaria endemic areas.

## Abstract 2

In a birth cohort study in Benin, Celia Dechavanne and colleagues examines associations between the IgG3-H435 variant, transplacental transfer of malaria-specific IgG3, and protection against Plasmodium falciparum malaria during infancy.

## Author summary

**Why was this study done?:**

- Human immunoglobulin G (IgG) is critical for immunity to many infections including malaria. The active transport of IgG across the placenta during pregnancy contributes to protection against malaria and other infections during early infancy.
- IgG is divided into 4 subclasses with different functions. IgG3 is unique among human subclasses in that it represents only 5% of total IgG, yet it is the most potent subclass for activating complement and cellular immune responses (opsonization). Its half-life in blood is 7 days, compared to 21 days for other subclasses.
- The potent opsonizing characteristics of IgG3 may contribute to protection against malaria, particularly during infancy as a result of transplacental transport of IgG from the mother.
- Recent studies have identified a single mutation in the binding domain of IgG3 that engages a receptor that regulates IgG persistence in blood and mediates IgG transport across the placenta.
- In this study, we examined whether women with this mutation had increased transplacental transfer of malaria-specific IgG3, and whether their offspring had a prolonged half-life of serum IgG3 and increased protection against malaria.

**What did the researchers do and find?:**

- We studied 497 pregnant women from Benin, west Africa, in a birth cohort study of their offspring, who were closely evaluated for the presence of malaria during the first year of life.
- We found that 24% of women had a specific mutation for IgG3 that was associated with significantly enhanced transplacental transfer and extended persistence of malaria-specific IgG3 in infant blood, compared to women who lacked the mutation.
- Offspring of women with this IgG3 mutation had a reduced risk of clinical malaria during infancy.

**What do these findings mean?:**

- This study provides evidence that transplacental transfer of malaria-specific IgG3 antibodies contributes to protection against malaria.
- The studied mutation in the IgG3 binding domain may have arisen to facilitate protection against malaria.
- Malaria vaccines that stimulate IgG3 production may demonstrate enhanced efficacy.

## Introduction

*Plasmodium falciparum* malaria remains a major cause of mortality and morbidity in children under 5 years of age in many endemic countries [1]. Death or severe disease typically arises from 1 to 3 overlapping syndromes: severe anemia, respiratory distress, or loss of consciousness [2]. Young infants under 6 months of age experience less clinical malaria compared to older children [3], but the mechanisms conferring immunity are not well understood [4]. One potential mechanism is active transplacental transfer of malaria-specific maternal immunoglobulin G (IgG) during gestation; however, the antigen targets and types of antibodies involved remain poorly defined [4]. Functional assays using cord blood plasma, such as antibody-dependent respiratory burst assays, show that immunoglobulins provide protection against severe malaria during the first 6 months of life [2]. This suggests that cytophilic immunoglobulin subclasses with potent opsonizing and complement fixing functions (e.g., IgG1 and IgG3) are important in protection against malaria. Recent studies have shown that malaria-specific IgG3 relative to other IgG subclasses is more strongly associated with malaria immunity (reviewed in [4]), and its passive transfer to the fetus may contribute to reduced risk of malaria in early infancy. IgG3 is notable compared to other IgG subclasses: it has higher affinity for complement component C1q and Fc gamma receptors (FcγRs), a 4-fold longer flexible hinge region, that are characteristics to enhance opsonization of malaria-infected erythrocytes and promotion of effector functions such as complement-dependent cytotoxicity, antibody-dependent cellular cytotoxicity, and respiratory burst phagocytosis [5,6]. In studies of malaria blood stage vaccine candidate antigens (e.g., GLURP), increased opsonic phagocytosis activity of IgG3 in vitro is strongly associated with reduced risk of febrile malaria, thus confirming the importance of IgG3 function in immunity to malaria [7].

Human IgGs are actively transferred across the placenta, with 30%–40% higher levels in newborn serum compared to maternal blood [8]. Preferential transport occurs for IgG1, followed by IgG4, IgG3, and IgG2 [9]. The neonatal Fc receptor (FcRn), expressed within endosomes in the syncytiotrophoblastic membrane, mediates IgG transfer by micropinocytosis. The interaction between the constant region of immunoglobulin heavy chain gamma (IGHG) and the FcRn is highly pH dependent. In particular, histidines at positions 310 and 435 (H310 and H435) on the IGHG Fc fragment become positively charged at pH 6.5, forming salt bridges with corresponding FcRn residues [10–12]. The FcRn is also a homeostatic receptor responsible for prolonging IgG half-life, by protecting it from lysosomal degradation and recycling it to systemic circulation [13]. Interestingly, IgG3 has a half-life of 7 days, compared to 21 days for IgG1 [14]. This difference in half-life has been attributed to the presence of arginine at position 435 (R435) in the IgG3 heavy chain instead of histidine (H435), as in IgG1, IgG2, and IgG4 [15]. In vitro studies demonstrate that IgG3-R435 has a lower binding affinity to FcRn [16]; consequently, IgG3-R435 is competitively inhibited by IgG1, resulting in reduced transplacental transfer and decreased half-life [5].

In contrast to other IgG subclasses, IgG3 is highly polymorphic, with distinct variants that give rise to allo-specific antibodies (allotypes). Three distinct IgG3 amino acid variants (IGHG3*17, IGHG3*18, and IGHG3*19), identified by allotypes G3m15 and G3m16, naturally contain a histidine at position 435. Both IgG3-H435 and IgG3-R435 may be present at different levels in the blood of heterozygous individuals [17]. The H435 variant is uncommon in Europeans (~1%), but is more prevalent in Asians (10%–25%) and in Africans (30%–60%) [18], suggesting it may be under positive selection in malaria endemic areas. The functional significance of this polymorphism for IgG3 transplacental transfer and IgG3 half-life, especially with respect to malaria, has not been much investigated [19]. Factors that impair or enhance transplacental transfer can alter the amount of pathogen-specific IgG in the newborn circulation and therefore susceptibility to infections, including malaria.

Here we examine the association between the IgG3-H435 polymorphism and transplacental transfer of *P*. *falciparum–*specific IgG1 and IgG3 in 497 Beninese mother–newborn pairs, and we investigate how this transfer is associated with the risk of malaria during infancy. Specifically, we explored the hypotheses that the maternal IgG3-H435 variant increases transplacental transfer of anti-malaria IgG3 to the newborn, prolongs IgG3 half-life in infant blood, and reduces the risk for malaria in infancy.

## Methods

### Ethics

The University of Abomey Calavi institutional review board (Benin) and the Consultative Ethics Committee of the Institute of Research for Development (France) approved the study protocol for the Beninese cohort. Before enrollment, all women signed an informed consent that also included consent for their children. All methods were carried out in accordance with approved guidelines.

### Study design and sample collection

Between 1 June 2007 and 31 July 2008, 572 newborns were enrolled in a birth cohort study in Tori-Bossito, in south Benin [20], an area classified as mesoendemic for malaria [21]. Infants were actively and passively followed for malaria until 12 months of age, as previously described [22]. After delivery, thick blood films were prepared from blood obtained by scraping the wall of the incision from the maternal side of the placenta. The presence of *P*. *falciparum* (placental malaria) was determined by microscopy. Active surveillance was conducted with weekly home visits, at which time axillary temperature and symptoms related to malaria were assessed. Febrile children were further evaluated at the local health center using a malaria rapid diagnostic test and a thick blood smear. Any child who developed symptoms of malaria at other times was encouraged to attend the health center, where a similar evaluation was performed and recorded (passive surveillance). Symptomatic (clinical) malaria was defined as axillary temperature > 37.5°C and positive blood smear for malaria, as previously described [22]. Symptomatic malaria was treated with artemether and lumefantrine combination therapy, as recommended by the National Malaria Control Program. Every month, during one of the weekly visits at home, blood smears were systematically collected to determine asymptomatic carriage (active surveillance). To increase the probability that febrile illness was due to malaria, only those individuals with >2,500 parasites/μl were included in the analysis of symptomatic malaria. This cutoff value was selected because few of the asymptomatic participants had parasitemia levels of >2,500 parasites/μl. Maternal venous and cord blood was obtained at delivery, and venous blood samples were collected from infants every 3 months. Plasma was separated and stored. Of the 572 participants initially enrolled in the study, 27 infants were excluded because of follow-up problems (11 with doubtful identification, 12 with extensive missing data, and 4 with missing individual malaria exposure) [20], and an additional 48 infants were excluded because they had missing placental malaria information and/or insufficient quantity or quality of DNA for genotyping, thus yielding 497 individuals available for analysis.

### Assessment of independent variables that could be associated with antibody transfer and malaria outcomes

Maternal hypergammaglobulinemia was defined as total maternal IgG of ≥1.6 g/dl. Gestational age was measured by the Ballard score [22], and premature birth was defined as birth at <37 weeks of gestation. Pregnant women were enrolled at delivery, when a questionnaire was administered to collect information on the women’s obstetric background and current pregnancy. Eighty-four percent of the pregnant women included in the study reported taking at least 1 dose of Fansidar (500 mg sulfadoxine and 25 mg pyrimethamine) for malaria chemoprophylaxsis [22]. Independent variables are listed in Table 1.

**Table 1:** Population characteristics according to the IgG3 polymorphism at position 435.

Characteristic	IgG3 polymorphism		*p*-Value
	R435 (*N =* 377)	H435 (*N =* 120)	
**Maternal factors**			* *
Placental malaria	39/374 (10.4%)	14/120 (11.7%)	0.703
Hypergammaglobulinemia (maternal total IgG ≥ 1.6 g/dl)	69/375 (18.4%)	18/119 (15.1%)	0.414
Bednet use	179/262 (68.3%)	58/79 (73.4%)	0.388
Malaria chemoprophylaxis use	309/375 (82.4%)	102/119 (85.7%)	0.399
Maternal weight (kilograms)	61.5 (±14.6)	61.1 (±13.1)	0.818
Maternal age (years)	27.5 (±5.5)	27.7 (±5.8)	0.739
Primiparous mother	316/377 (83.8%)	104/120 (86.6%)	0.453
**Newborn factors**			
Male	191/375 (50.9%)	56/119 (47.1%)	0.562
Gestational age (weeks)	38.5 (±1.7)	38.4 (±1.9)	0.791
Term birth (≥37 weeks)	339/375 (90.4%)	103/119 (86.6%)	0.234
Birth weight (grams)	4,007.3 (±551.0)	4,052.3 (±459.8)	0.425

Data given as *n/N* (percent) or mean (SD). R435 represents individuals homozygous for IgG3-R435 (*N =* 377). H435 represents individuals heterozygous (*N =* 117) or homozygous (*N =* 3) for IgG3-H435. Pearson chi-squared tests were used to compare percentages, and *t* tests were used to compare means.

### Individual malaria exposure

The homes of all infants were localized by GPS. Mosquitoes were collected at 4 houses in each study village (*N =* 9 villages) over 3 successive nights, every 6 weeks between 1 July 2007 and 31 July 2009. Rainfall was recorded twice a day with a pluviometer in each village during the entire follow-up [20,23]. In the analysis, the number of rainy days during the 10 days before a mosquito catch was considered. The following data were also collected during each catch: season, type of soil 100 meters around the house, presence of small pools of water from partial/abandoned construction 100 meters around the house, presence of a watercourse within 500 meters around the house, normalized difference vegetation index 100 meters around the house, ownership of bednets, use of insect repellent, and number of inhabitants per house. Based on these variables, a predictive regression model was developed, and an individual malaria exposure variable was constructed for use in this analysis. Details of this model are published elsewhere [20,23].

### Antibody measurement by enzyme-linked immunosorbent assay (ELISA)

ELISA was performed on maternal, cord, and infant sera as described previously [24]. The concentrations of total human IgG and specific IgG1 and IgG3 directed at promising blood stage vaccine candidate antigens were measured. Antigens for study included apical membrane antigen 1 (AMA1), merozoite surface protein 1–19 (MSP1_19_), MSP2 (2 allelic families, 3D7 and FC27), MSP3, and glutamate-rich protein (GLURP; 2 regions, R0 and R2), as detailed previously (supplementary information in [24]). A dedicated program in R statistical software [25] was derived from ADAMSEL FLP b039 (http://www.malariaresearch.eu/content/software) and was used to analyze antibody concentrations by ELISA optical density as described previously [24]. Briefly, censored values (below detection threshold or over saturation) were imputed using a (log) linear regression model, taking into account confounding variables. The model was fitted using a stochastic expectation maximization algorithm and was robust since the association between imputed and measured values was very good (*R*^2^≥0.91) [24].

### Determination of maternal IgG3 polymorphism by sequencing

Maternal DNA was extracted with a Qiagen kit according to manufacturer recommendations (Qiagen, Valencia, CA, US). At least 1 ng/μl DNA was amplified with a forward primer (FWD 5′-GTCGGGTGCTGACACATCTG-3′) and a reverse primer extended by a universal primer M13 (REV 5′-AGCGGATAACAATTTCACACAGGA | CTTGCCGGCYRTSGCACTC-3′) [26]. The additional M13 sequence extended the amplicon size in order to enhance sequence quality. The amplification was performed with AccuPrime Taq DNA Polymerase, High Fidelity (Fisher Scientific, Florence, KY, US) and the buffer II (delivered with the enzyme). PCR product purification was performed with the Wizard SV 96 PCR Clean-Up System (Promega, Madison, WI, US). For the sequencing, the amplification forward (FWD) and/or a second forward (FWD2 5′-AGGTCAGCCTGACCTGCCTG-3′) primer [26] was used for sequence accuracy and led to 805- and 277-nucleotide-long sequences, respectively (from bp 1,382 or 1,910 to bp 2,186; accession number NC_000014.9). In case of a nonconclusive sequencing, a third reverse primer was used (M13-REV 5′-AGCGGATAACAATTTCACACAGGA-3′). The sequencing was performed by Eurofins Genomics (Louisville, KY, US).

### Statistical analyses

Prespecified confounding factors were maternal malaria at delivery, hypergammaglobulinemia, bednet use, malaria chemoprophylaxis during pregnancy, maternal weight and age, parity, newborn sex, gestational age, and birth weight. These factors were evaluated with respect to the IgG3-H435 polymorphism by Pearson chi-squared test (to test the difference between proportions) or Student unpaired *t* test (to test the difference between means) as appropriate.

The transfer of malaria-specific antibodies is defined by the cord-to-mother transfer ratio (CMTR, cord IgG level divided by maternal IgG level). The CMTR was used in the different analyses as dichotomized above and below the median. A univariate logistic regression was performed to compare the CMTR for IgG1 and IgG3 for all mothers, stratified by R435H polymorphism. Given the multiple comparisons in this analysis, significant difference was considered as *p ≤* 0.007 after a Bonferroni correction was applied. A multivariate logistic regression model assessed the association of the IgG3-H435 polymorphism with the degree of transplacental transfer, adjusted for the potentially confounding variables described above. The competition between IgG1 and IgG3 for FcRn binding was tested by a multivariate logistic regression model, after stratification by maternal hypergammaglobulinemia. In this model, the dependent variable was the CMTR of malaria-specific IgG3, and the independent variables were malaria-specific maternal IgG1 level and the confounding variables listed below. The half-life of IgG3-H435 (versus IgG3-R435) in infant blood was evaluated by a mixed linear regression analysis using data from 302 infants for whom antibody levels at birth (cord blood) and 3 and 6 months of age were available, and for whom no evidence of a rise in malaria-specific antibody levels between 0 and 3 months or between 3 and 6 months of age was noted. A hierarchical model was used to account for multiple monthly measurements on the same individuals. The association between protection against malaria, defined as time to first malaria parasitemia, and the transferred IgG subclasses was illustrated by Kaplan–Meier curves and evaluated by log-rank test. A multivariate Cox analysis was performed to assess the association between the delay to first symptomatic malaria over the first year of life and the IgG3-H435 polymorphism. In this multivariate analysis, exposure to *P*. *falciparum* infection (as described above [23]) was used as an independent variable as were the other variables listed below. A Poisson regression was performed with the same independent variables as used for the multivariate Cox regression to evaluate the relationship of maternal IgG3-H435 (versus IgG3-R435) and the number of symptomatic malaria and asymptomatic malaria episodes between birth and 12 months of age. All multivariate analyses included the variables with *p* ≤ 0.20 in the univariate step. Statistical significance was set at *p <* 0.05. The variance inflation factor (VIF) values were tested in models where a collinearity could be suspected. All statistical analyses were performed using Stata, version 13.0 (StataCorp, College Station, TX, US). The original protocol and modifications are available in S1–S3 Protocols.

## Results

The overall frequency of the IgG3-H435 variant in the population group of Tori-Bossito, south Benin, was 0.12 compared to 0.88 for IgG3-R435. A total of 117 mothers were heterozygous and 3 were homozygous for IgG3-H435, resulting in 24% of study participants (pregnant women) carrying the allele (Table 1). The IgG3-H435 variant was not associated with prespecified confounding factors likely to influence transplacental antibody transfer and/or malaria risk in mothers and infants (Table 1).

### Do mothers with IgG3-H435 have a higher transplacental transfer of malaria-specific IgG3?

We explored the hypothesis that the maternal IgG3-H435 variant increased transplacental transfer of anti-malaria IgG3 to the newborn: it was expected that (i) the maternal IgG3-H435 variant would be associated with a better transplacental transfer of IgG3 and (ii) this IgG3 transfer would be equivalent to IgG1 transfer, for which the variant is always IgG1-H435.

**Fig 1:**
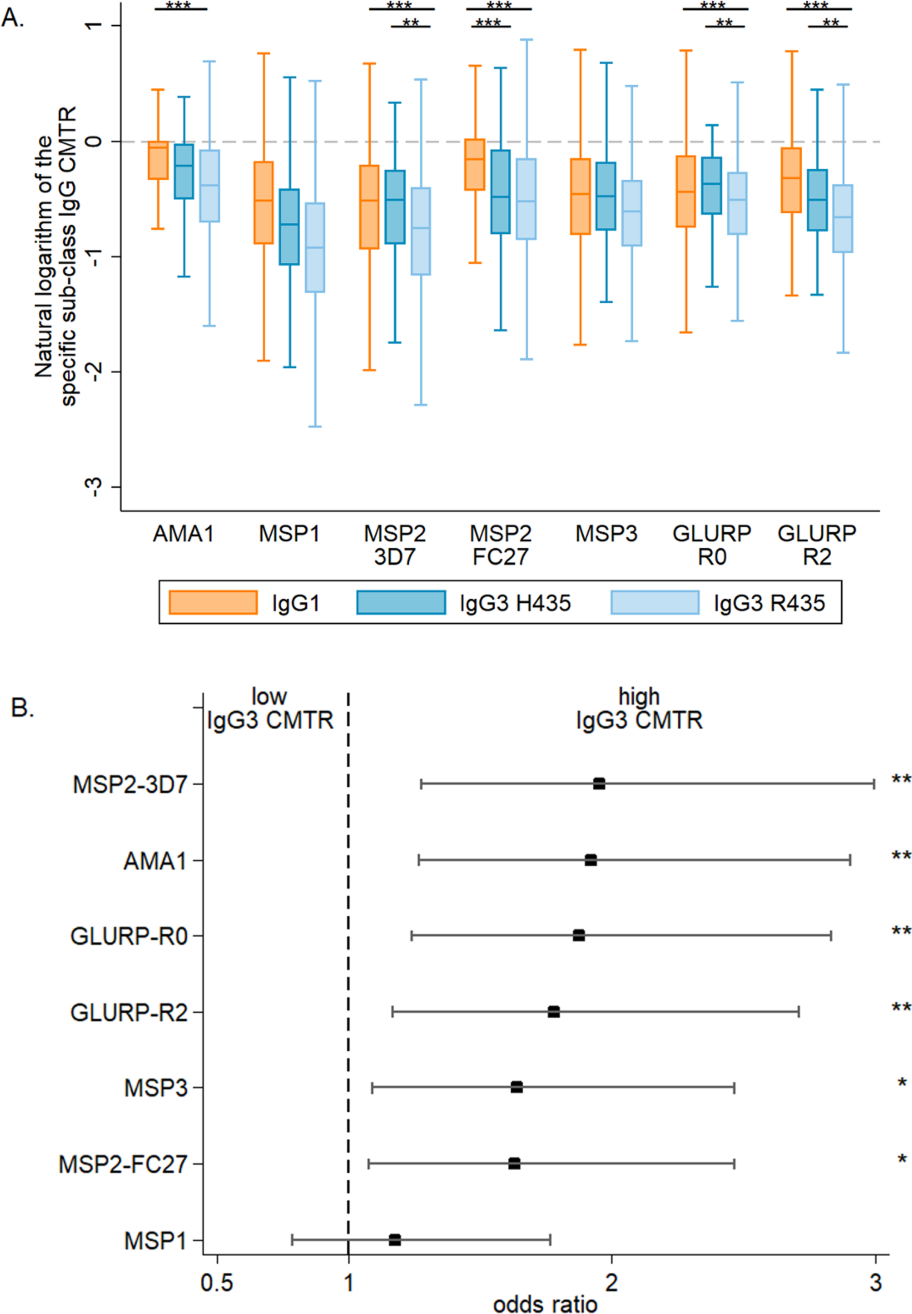
The cord-to-mother transfer ratio of malaria-specific IgG1, IgG3-R435, and IgG3-H435. (A) The cord-to-mother transfer ratios (CMTRs) are represented: malaria-specific IgG1 (orange bars), IgG3 for women with the IgG3-H435 allele (dark blue bars, *N =* 120), and IgG3 for women homozygous for the IgG3-R435 allele (light blue bars, *N =* 377). Statistical comparison with IgG1 was performed for 377 women homozygous for IgG3-R435 and 120 women with the IgG3-H435 allele. Data are shown as box and whisker plots, and the *p-*values of the logistic regression univariate analysis are represented as follows: ** *p* ≤ 0.007, *** *p* ≤ 0.001. Only *p-*values ≤ 0.007 were considered significant after Bonferroni correction for multiple comparisons. (B) A multivariate logistic regression was performed to test whether individuals with IgG3-H435 had greater CMTR (over or below the median) relative to those with only IgG3-R435. The association is represented by the odds ratio (± 95% confidence interval) for each malaria antigen. Adjusted variables are shown in Table 1. There was no collinearity between all tested variables, and variance inflation factor (VIF) values were ≤ 1.08.

Overall, malaria-specific IgG1 and IgG3 levels were similar in mothers and neonates (S1 Fig). The transplacental transfer of malaria-specific IgG3 that recognized MSP2-3D7, GLURP-R0, and GLURP-R2 was significantly greater for mothers with IgG3-H435 compared to those with homozygous IgG3-R435 (Fig 1A). A similar pattern was observed for the other antigens, although these differences were not significant. Using multivariate analysis to adjust for other factors that could influence transplacental transfer (see Table 1), we found that transfer of anti-malarial IgG3 was 60% to 95% higher for IgG3-H435 than for homozygous IgG3-R435 (Fig 1B, all antigens *p <* 0.01 except MSP1_19_). Of note, transplacental transfers of IgG3-H435 and IgG1 were similar for many of the antigens (Fig 1A), except for MSP2-FC27 (*p <* 0.01). For a subset of the antigens tested, other factors were independently associated with reduced transplacental transfer of malaria-specific IgG3, including the presence of placental malaria, increased maternal malaria-specific IgG3 levels, and increased levels of maternal total IgG (Table 2).

**Table 2:** Association between cord-to-mother ratio (CMTR) and factors that could influence transplacental transfer: Placental malaria, maternal total IgG, and maternal malaria-specific IgG3.

Antigen	Factor	*N*	OR	95% CI	*p-*Value
**AMA1**	Presence of placental malaria	483	0.76	0.40, 1.32	0.362
	Ln maternal specific IgG3		0.95	0.88, 1.03	0.195
** **	Ln maternal total IgG		0.98	0.80, 1.19	0.810
**MSP1**_**19**_	Presence of placental malaria	492	0.80	0.41, 1.36	0.449
	Ln maternal specific IgG3		**0.86**	**0.80, 0.93**	**<0.001**
** **	Ln maternal total IgG		0.94	0.79, 1.18	0.539
**MSP2-3D7**	Presence of placental malaria	475	0.83	0.43, 1.44	0.513
	Ln maternal specific IgG3		**0.89**	**0.80, 0.98**	**0.021**
** **	Ln maternal total IgG		**0.69**	**0.56, 0.86**	**0.001**
**MSP2-FC27**	Presence of placental malaria	489	1.04	0.50, 1.66	0.876
	Ln maternal specific IgG3		0.92	0.85, 1.06	0.127
** **	Ln maternal total IgG		**0.80**	**0.65, 0.97**	**0.026**
**MSP3**	Presence of placental malaria	483	0.87	0.47, 1.53	0.618
	Ln maternal specific IgG3		0.95	0.89, 1.04	0.224
** **	Ln maternal total IgG		0.92	0.72, 1.07	0.421
**GLURP-R0**	Presence of placental malaria	481	**0.52**	**0.29, 0.98**	**0.039**
	Ln maternal specific IgG3		**0.88**	**0.81, 0.96**	**0.002**
** **	Ln maternal total IgG		0.87	0.71, 1.06	0.149
**GLURP-R2**	Presence of placental malaria	494	**0.48**	**0.26, 0.91**	**0.023**
	Ln maternal specific IgG3		**0.83**	**0.76, 0.91**	**<0.001**
** **	Ln maternal total IgG		**0.76**	**0.62, 0.94**	**0.037**

The odds ratios (ORs) were calculated based on the dichotomized CMTR (above or below the median). There was no collinearity between all the tested variables, and the variance inflation factor (VIF) values were all ≤1.08. In bold: results with *p* < 0.05.

### Are high levels of maternal anti-malaria IgG1 associated with decreased transplacental transfer of anti-malaria IgG3?

Prior studies have shown that, in vitro, IgG1 (always H435) competes with IgG3-R435 for binding to FcRn [5]. To examine whether this occurs in vivo, serum samples obtained from mothers at birth were assayed for level of malaria-specific GLURP-R2. Women with GLURP-R2-specific IgG1 above the median compared to those below the median demonstrated a 62% reduced transplacental transfer of GLURP-R2-specific IgG3 (odds ratio [OR] = 0.58 [95% CI 0.39, 0.87], *p =* 0.008, *N =* 405). This analysis was performed in a subset of women without hypergammaglobulinemia (<1.6 g/dl of total IgG, *N =* 405) because elevated IgG is an independent risk factor for impaired transplacental transport of IgG3 (Table 2). The competition between IgG1 and IgG3 for binding to FcRn and therefore for transplacental transfer is represented by the association between a high level of maternal IgG1 and a decreased transfer of IgG3. We noted a greater competition for transplacental transfer of IgG3 for GLURP-R2 in women with homozygous IgG3-R435 compared to IgG3-H435 (OR = 0.54 [95% CI 0.21, 1.43], *p =* 0.22), but this difference was not statistically significant. For the other antigens, the level of maternal anti-malaria IgG1 was not associated with decreased transplacental transfer of IgG3.

### Does transplacentally transferred IgG3-H435 persist longer in early infancy compared to IgG3-R435?

**Fig 2:**
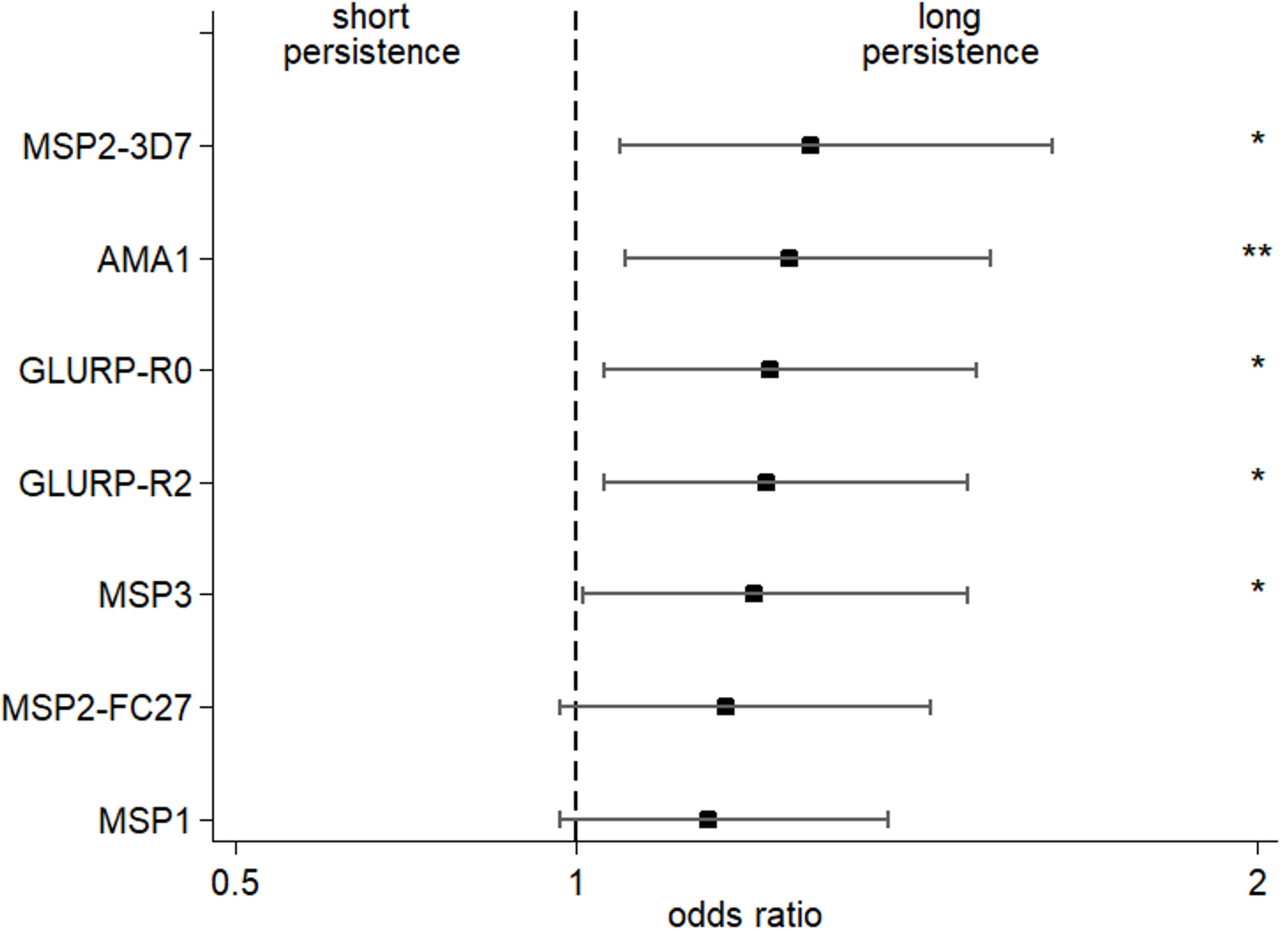
Increased persistence of maternally derived malaria-specific IgG3 among young infants born to women with the IgG3-H435 allele compared to those homozygous for IgG3-R435. The odds ratio (± 95% confidence interval) of maternal malaria-specific IgG3-H435 persistence at 6 months relative to homozygous IgG3-R435 based on dichotomized cord-to-mother transfer ratio (over or below the median) using multivariate regression analysis adjusted for the variables shown in Table 1. To reduce the possibility that IgG3 was produced by malaria-infected infants, samples from any infant showing an increase in malaria-specific IgG3 between 0 and 3 months or between 3 and 6 months of age (any antigen) were removed from the analysis (*N =* 195). The odds ratio was obtained after adjustment for malaria-specific IgG3 in maternal peripheral blood (see Table 3). Of 302 infants, between 3 and 11 had missing data (according to the tested antigen) and were not included in the analysis (see details in Table 3). There was no collinearity between all tested variables, and variance inflation factor (VIF) values were all ≤2.17.

For mothers with high levels of malaria-specific IgG3 at birth, we detected higher levels of malaria-specific IgG3 in their infants, and for a longer period of time (Table 3). After adjustment for maternal IgG3 level, we found that offspring of mothers with IgG3-H435 had a 28% to 35% greater persistence of malaria-specific IgG3 between birth and 6 months of age, compared to offspring of mothers homozygous for IgG3-R435 (Fig 2). This difference was significant for AMA1, MSP2-3D7, MSP3, GLURP-R0, and GLURP-R2, but not for MSP1_19_ and MSP2-FC27.

**Table 3:** Persistence of malaria-specific IgG3 at 6 months of age related to level of maternal anti-malaria IgG3.

Antigen	*N*	Odds Ratio	95% CI	*p-*Value
AMA1	291	2.52	2.41, 2.64	<0.001
MSP1_19_	298	2.46	2.38, 2.56	<0.001
MSP2-3D7	292	2.36	2.24, 2.48	<0.001
MSP2-FC27	289	2.33	2.20, 2.46	<0.001
MSP3	292	2.48	2.38, 2.57	<0.001
GLURP-R0	294	2.36	2.25, 2.46	<0.001
GLURP-R2	294	2.38	2.28, 2.49	<0.001

Comparing the groups IgG3-H435 and IgG3-R435, a longer persistence of anti-malaria IgG3 is defined as a higher level of antibody at 6 months of age. Infants with malaria-specific IgG3 boost between 0 and 3 months or between 3 and 6 months of age (any antigen) were removed from the analysis. Out of 302 infants; the effective sample size for each model of is indicated by the *N*. There was no collinearity between all the tested variables: the final models were all tested, and the variance inflation factor (VIF) values were all ≤2.17.

### Is an increased transplacental transfer of malaria-specific IgG3 associated with a reduced risk of malaria in early infancy?

**Fig 3:**
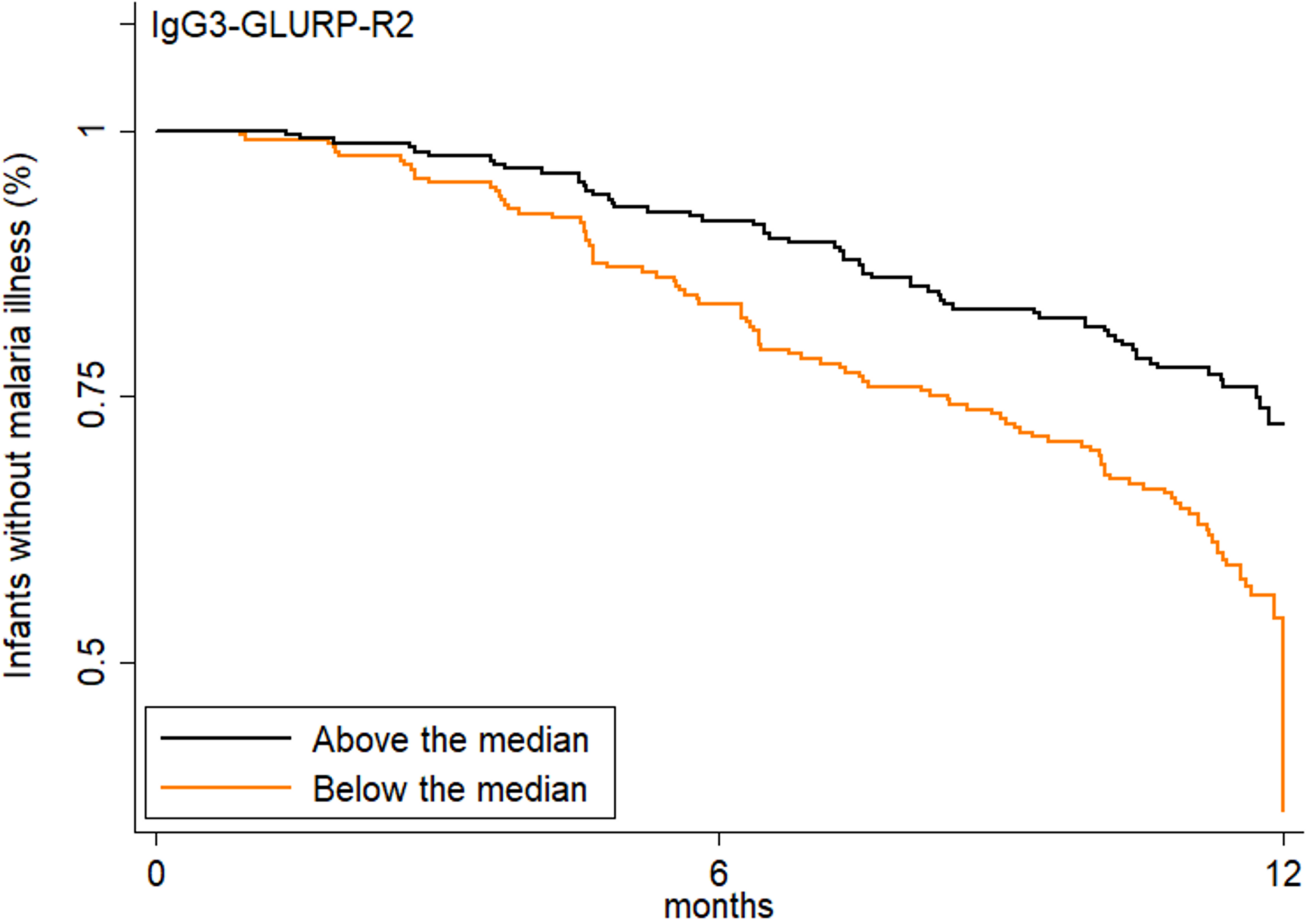
Increased transplacental transfer of GLURP-R2-specific IgG3 is associated with delayed time to first symptomatic malaria. Dichotomized cord-to-mother transfer ratio (above or below the median; *N =* 493 infants). The log-rank analysis yielded *p <* 0.001, and the Cox proportional hazard analysis model adjusted for malaria exposure and placental malaria gave a hazard ratio of 0.59 (95% CI 0.45, 0.77, *p <* 0.001).

Prior studies have shown that antibody responses directed against GLURP, especially block 2 region, are associated with a reduced risk of clinical malaria in different populations [7,27]. In the present study, an increased transplacental transfer of GLURP-R2-specific IgG3 was associated with a delay in time to first symptomatic malaria in the first year of life (Fig 3; hazard ratio [HR] adjusted for maternal placental malaria and infant malaria exposure = 0.59 [95% CI 0.45, 0.77], *p <* 0.001). Similar results were observed for GLURP-R0 (HR = 0.73 [95% CI 0.56, 0.95], *p =* 0.02), but not for the other malaria antigens (S1 Table). This suggests that IgG3 directed to GLURP has greater functional activity in blood stage malaria infection.

### Do offspring of IgG3-H435 mothers have a lower risk of malaria during infancy?

**Fig 4:**
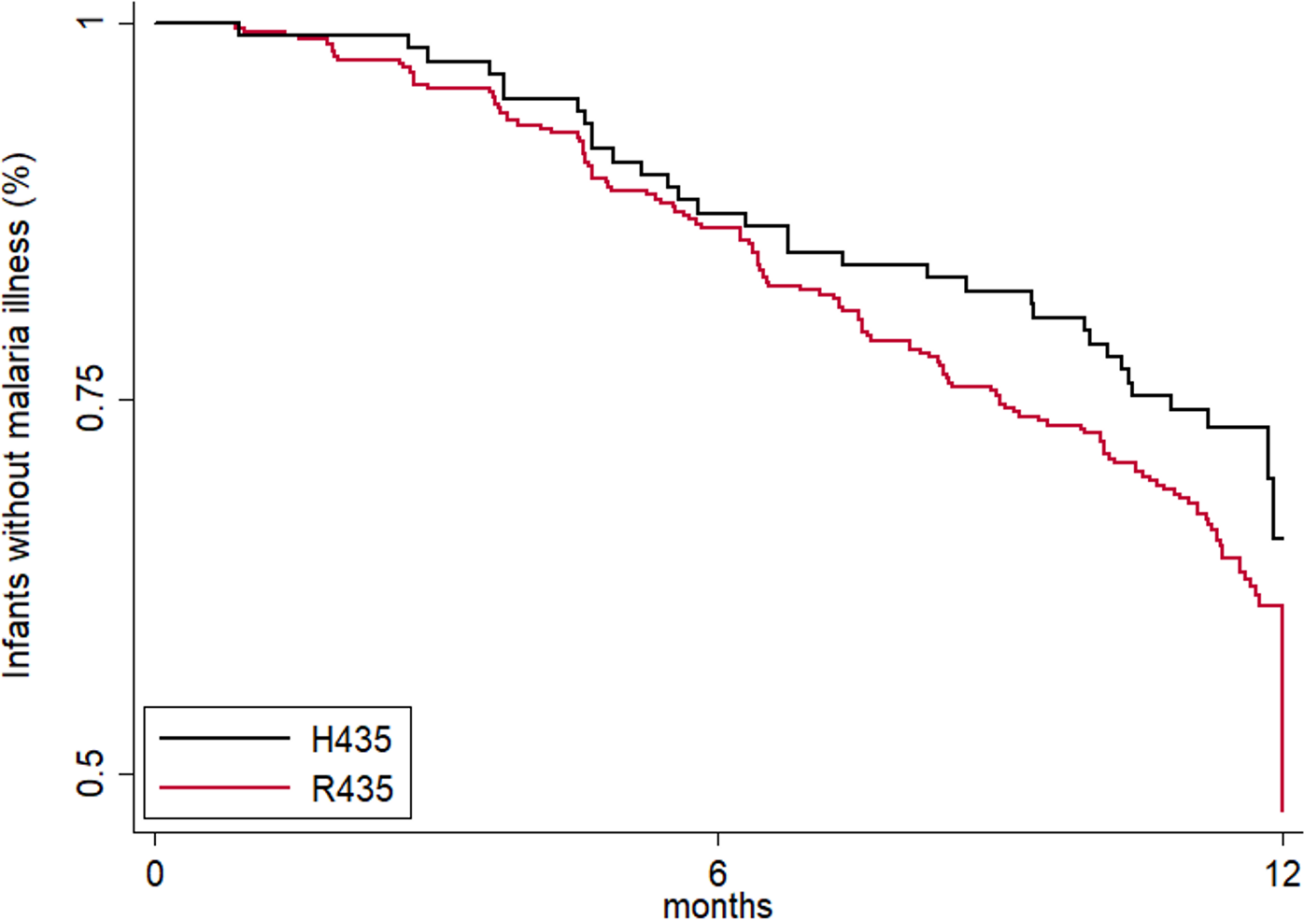
Maternal IgG3-H435 polymorphism is associated with a decreased risk of symptomatic malaria in infants from birth to 12 months of age. The time to first symptomatic malaria episode during infancy is shown. The log-rank analysis yielded *p =* 0.102. The Cox proportional hazard analysis adjusted for malaria exposure and placental malaria gave a hazard ratio of 0.69 (95% CI 0.46, 1.05, *p =* 0.083).

Because women with IgG3-H435 have increased transplacental transfer of IgG3 and their offspring have IgG3 with prolonged half-life during infancy, we examined whether offspring of mothers with IgG3-H435 versus homozygous IgG3-R435 had a reduced risk of malaria in infancy. Using Cox proportional hazard analysis (Fig 4), there was a delay in time to first symptomatic (clinical) malaria infection as defined by a fever of >37.5°C and >2,500 parasites/μl among offspring of women with IgG3-H435 versus homozygous IgG3-R435 (HR = 0.69 [95% CI 0.37, 1.03], *p =* 0.08, adjusted for individual malaria exposure and placental malaria), but this difference was not statistically significant. The difference was most noticeable at 6–8 months of age, when survival curves diverged. To further explore the relationship of IgG3-H435, infant age, and susceptibility to malaria, we examined the data using a Poisson regression model stratified by 0–6 versus 6–12 months of age and whether the infant had clinical malaria or asymptomatic parasitemia (Table 4). Of note, this analysis was not prespecified in the initial protocol. Offspring of women with the IgG3-H435 allele had a 32% reduced risk (incidence rate ratio [IRR] = 0.68 [95% CI 0.51, 0.91], *p =* 0.01) of clinical malaria during infancy (0–12 months). This association was most pronounced among infants 6–12 months of age (IRR = 0.61 [95% CI 0.43, 0.87], 39% reduced risk, *p =* 0.007). There was no association between the IgG3-H435 allele and the risk of asymptomatic malaria (Table 4). Of note, altering the definition of clinical malaria using different parasitemia levels, >5,000/μl, >10,000/μl, etc., does not alter the association with protection; however, the confidence interval widens because the sample size diminishes.

**Table 4:** The cumulative number of malaria infections recorded from birth to 12 months of age.

Malaria type	0–12 months				0–6 months				6–12 months			
	R435, *N* (rate)	H435, *N* (rate)	IRR [CI]	*p-*Value	R435, *N* (rate)	H435, *N* (rate)	IRR [CI]	*p-*Value	R435, *N* (rate)	H435, *N* (rate)	IRR [CI]	*p-*Value
Symptomatic malaria	205 (0.54)	44 (0.36)	0.68 [0.51, 0.91]	**0.010**	55 (0.15)	14 (0.12)	0.88 [0.53, 1.47]	0.631	150 (0.40)	30 (0.25)	0.61 [0.43, 0.87]	**0.007**
Asymptomatic malaria	157 (0.42)	44 (0.34)	0.95 [0.68, 1.32]	0.760	58 (0.15)	22 (0.18)	1.16 [0.70, 1.91]	0.570	99 (0.26)	27 (0.22)	0.83 [0.53, 1.28]	0.404

Women homozygous for IgG3-R435, *N =* 377; women with IgG3-H435, *N =* 120. Incidence rate ratios (IRRs) adjusted for placental malaria infection and individual malaria exposure. Symptomatic malaria defined as fever > 37.5°C and >2,500 parasites/μl of blood. In this model (0–12 months), placental malaria was associated with a 52% increased risk of clinical malaria (incidence rate ratio [IRR] = 1.52 [95% CI 1.05, 2.19], *p =* 0.025), and infant malaria exposure was associated with an 8% increased risk of clinical malaria (IRR = 1.08 [95% CI 1.06, 1.11], *p <* 0.001). *p-*Values < 0.05 shown in bold.

## Discussion

Human IgG is the only antibody isotype that is actively transferred across the placenta, providing passive immunity for the newborn. With respect to all IgG subclasses, IgG3 is noteworthy for its shorter half-life and diminished capacity for transplacental transfer. Due to an arginine substitution for histidine at position 435, found exclusively in IgG3, binding to FcRn is reduced [5]. Why IgG3 differs from other IgG subclasses in several respects is unclear. One possibility is that by limiting the transplacental transfer of maternal IgG3 directed to fetal antigens, the risk of fetal alloimmune pathology is reduced [28]. Also, the potent pro-inflammatory properties of IgG3 could be tempered by its shorter half-life [29]. Yet the powerful effector function of IgG3 may be beneficial against some pathogens such as *P*. *falciparum*, where malaria-specific cytophilic IgGs have been most strongly associated with protection [7,30]. Since infants are most susceptible to malaria infection, enhanced transplacental transfer of IgG3 with prolonged half-life may be advantageous. In support of this hypothesis, we showed that 24% of Beninese women living in an area highly endemic for malaria possess the IgG3-H435 allele, which enhances IgG3 binding to FcRn [5]. We demonstrated that women with this allele have increased transplacental transfer of malaria-specific IgG3 to their fetus, and this IgG3 persists longer in infant blood than IgG3-R435. We showed that increased transplacental transfer of IgG3 directed to the malarial GLURP antigen is strongly associated with reduced risk of clinical malaria, and offspring of mothers with the IgG3-H435 allele have reduced risk of clinical malaria in infancy compared to offspring of women with homozygous IgG3-R435. Together, these data support the conclusion that malaria-specific IgG3 contributes to protection against clinical malaria during infancy.

To our knowledge, this is the first study to show that an antigen-specific IgG3-H435 amino acid polymorphism influences transplacental transfer of IgG3, prolongs its half-life, and is associated with protection against a major pathogen in vivo. Prior studies have shown extended persistence of IgG3-H435 compared to IgG3-R435 in agammaglobulinemic patients who received intravenous IgG replacement [5]. Other studies demonstrated, using an ex vivo placental perfusion model [6], that IgG3-H435 transferred as efficiently as IgG1 across the human placenta; this was confirmed in vivo in 6 healthy pregnant Chinese women with the G3m16+ allotype, which enabled expression of IgG3-H435 [19]. Thus, our work confirms and expands the observation that IgG3-H435 alters the biological characteristics of IgG3 by enhancing its transplacental transfer and prolonging its half-life in vivo.

Offspring of women with IgG3-H435 had a 31% to 39% reduced risk of clinical malaria during infancy based on 2 independent analytic models. There was no association with protection against asymptomatic malaria, suggesting that the association was not related to exposure, but to the ability to control the level of parasitemia. This association was most pronounced at 6 to 8 months of age, when most of the passively transferred antibody protection in an infant has typically waned. There are several possible explanations for a delayed protective effect. First, IgG3-H435 may persist longer than other IgG subclasses to extend the period of passive protection. This is supported in studies of X-linked agammaglobulinemic individuals treated with intravenous IgG from donors with IgG3-H435, where IgG3-H435 antibody persisted 30% longer than that of the other subclasses in vivo [5]. Second, malaria-specific IgG1 may mask the protective effect of IgG3-H435 early on, but once the more abundant IgG1 has bound and cleared malaria antigen, the persistent effect of IgG3-H435 is relatively more important. Third, offspring of women with IgG3-H435 are more likely to have inherited the variant themselves, which may contribute to stronger acquisition of natural immunity in the infant.

Levels of cytophilic antibody to merozoite surface antigens (i.e., levels of IgG1 and IgG3) often correlate more strongly with protection against malaria than levels of IgG from the other subclasses. Thus, an enhanced association with protection against malaria would be expected for higher levels of functional antibodies directed against blood stage antigens [7,31–33]. The most important characteristics of functional antibodies include the capacity to activate complement and the ability to opsonize merozoites or malaria-infected erythrocytes for uptake by phagocytic cells. Of the IgG subclasses, IgG3 exemplifies these characteristics in that it has the greatest ability to activate complement and the highest affinity for FcγRs [34]. Thus, a mutation enhancing transplacental transfer of IgG3 and prolonging its half-life may be under positive selection in malaria endemic areas, where infant mortality due to malaria can reach 20% [1]. Supporting this possibility is the observation that greater frequencies of the SNP rs4042056 that encodes IgG3-H435 have been recorded in populations where malaria historically is more frequent (e.g., South Asian and African, ~0.12), as compared to northern European populations (~0.01) [18,35]. More information regarding IGHG3 polymorphism frequencies in endemic populations will help to clarify potential selective pressures on this SNP and its role in malaria clinical outcomes.

Almost all of the women with the IgG3-H435 haplotype in the study were heterozygotes. Expression of immunoglobulin genes undergoes allelic exclusion in individual B cells, as only 1 IgG3 heavy chain is expressed in a specific B cell [36]. Thus, throughout clonal selection, B cell proliferation, and memory B cell formation, all progeny of an individual B cell with activity against a given malarial epitope express the same IgG3 heavy chain. However, other pre-B cells undergoing a different IgG recombination could retain a different IgG3 heavy chain allele. This would result in malaria-specific B cells expressing IgG3 with either H435 or R435 alleles, although since they would arise from different B cell precursors, they may not recognize the exact same epitopes. The presence of both IgG3-H435 and IgG3-R435 in the serum of a single individual has been confirmed by mass spectroscopy of IgG3 from individuals with the IgG3-H435 allele [17]. However, in any one person there may be quite different levels of IgG3-H435 and IgG-R435 to specific antigens. Thus, in the current study, individuals bearing the IgG3-H435 haplotype would have, on average, just half of their IgG3 with H435 heavy chain, thus diluting a potentially protective effect.

Studies examining immune correlates of protection against malaria are frequently hampered by the inability to adequately control for exposure. In this investigation, frequent active and passive surveillance for malaria and a detailed assessment of malaria exposure at the individual level mitigate this limitation. Moreover, the observation that a single point mutation of IgG3 can measurably be associated with transplacental transfer of IgG3 to the newborn and extend its half-life, independent of other risk factors for malaria, allows for a more precise assessment of the role of malaria-specific IgG3 in protecting against malaria. There are limitations to the study as well. Almost all of the women carrying the IgG3-H435 allele were heterozygous, and we could not assess how much malaria-specific IgG3-H435 relative to IgG3-R435 was present in a given individual. In addition, the proportion of malaria-specific IgG produced by infants relative to that acquired from their mothers was not assessed, and how the relative proportions contribute to protection during infancy remains undefined. Finally, we could not determine the genotypes of the infants with respect to IgG3-H435 because requisite DNA was unavailable.

In conclusion, our study shows that the IgG3-H435 variant can have a profound association with transplacental transfer of malaria-specific IgG3, and it prolongs IgG3 half-life in infant blood, thereby enhancing immunity to malaria in infancy. This suggests that cytophilic IgG3, with a longer half-life following exposure to *P*. *falciparum*, may be of critical importance in protection against malaria. Vaccine approaches whereby malaria-specific IgG3 antibody responses are enhanced may prove to be especially useful.

## Supporting information

S1 Checklist STROBE checklist. (DOC)

S1 Fig IgG subclass levels to 7 malaria antigens between mothers and their offspring. The log-transformed concentrations of IgG1 and IgG3 to malaria antigens are presented in this figure. Maternal levels are in gray and newborn levels are in white.
(TIF)

S1 Protocol Complete version of the original protocol in French. (DOC)

S2 Protocol Summarized version of the original protocol in English. (DOCX)

S3 Protocol Modifications from the original protocol in English. (DOCX)

S1 Table Association between the transfer of specific IgG3 and symptomatic malaria over the first year of life. (XLSX)

## Abbreviations

CMTR: cord-to-mother transfer ratio
ELISA: enzyme-linked immunosorbent assay
FcRn: neonatal Fc receptor
IgG: immunoglobulin G
HR: hazard ratio
IGHG: immunoglobulin heavy chain gamma
IRR: incidence rate ratio
OR: odds ratio
VIF: variance inflation factor
