# Supplementary material for: Associations between an IgG3 polymorphism in the binding domain for FcRn, transplacental transfer of malaria-specific IgG3, and protection against Plasmodium falciparum malaria during infancy: A birth cohort study in Benin
Source: PLoS Med. 2017 Oct 9;14(10):e1002403. doi: 10.1371/journal.pmed.1002403 (PMC5633139; doi:10.1371/journal.pmed.1002403)

STROBE Statement—Checklist of items that should be included in reports of ***cohort studies***

|  | Item No | Recommendation |
| --- | --- | --- |
| **Title and abstract** | 1 | (a) Associations between an IgG3 polymorphism in the binding domain for FcRn, transplacental transfer of malaria-specific IgG3, and protection against Plasmodium falciparum malaria during infancy: A birth cohort study in Benin |
|  |  | (*b)* Abstract |
| Introduction | | |
| Background/rationale | 2 | Abstract and Introduction |
| Objectives | 3 | Introduction |
| Methods | | |
| Study design | 4 | Method – Study design and sample collection |
| Setting | 5 | Method – Study design and sample collection |
| Participants | 6 | (*a*) Method – Study design and sample collection |
|  |  | (*b*) Between 0-12 months of age, 249 symptomatic malaria and 201 symptomatic malaria were detected. |
| Variables | 7 | Method - Assessment of independent variables |
| Data sources/ measurement | 8* | Method - ELISA and genotyping paragraphs |
| Bias | 9 | Describe any efforts to address potential sources of bias |
| Study size | 10 | Of the 572 participants initially enrolled in the study, 27 infants were excluded because of follow-up problems (11 with doubtful identification, 12 with extensive missing data, and 4 with missing individual malaria exposure), and an additional 48 infants were excluded because they had missing placental malaria information and/or insufficient quantity or quality of DNA for genotyping, thus yielding 497 individuals available for analysis. |
| Quantitative variables | 11 | The transfer of malaria-specific antibodies is defined by the cord-to-mother transfer ratio (CMTR, cord IgG level divided by maternal IgG level). The CMTR was used in the different analyses as dichotomized above and below the median. |
| Statistical methods | 12 | (*a*) Method - Statistical analyses |
|  |  | (*b)* Method - Statistical analyses |
|  |  | (*c*) Infant with missing data were excluded from the analysis |
|  |  | (*d*) Infant with missing data were excluded from the analysis |
|  |  | (*e*) Describe any sensitivity analyses: not applicable |
| Results | | |
| Participants | 13* | (a) 572 mother/infant pairs were potentially eligible, 497 were confirmed eligible and included in the study with a complete follow-up. Depending on the analysis, the number of mother/infant pairs analysed varies (see below, the flow diagram) |
|  |  | (b) Infant with missing data were excluded from the analysis |
|  |  | (c) See the flow diagram after the checklist. |
| Descriptive data | 14* | (a) Table 1 |
|  |  | (b) See the flow diagram after the checklist. |
|  |  | (c) 493 infants have a complete follow-up. The 4 infants with information only at birth were excluded for the analysis taking into account the follow-up information (the 3 last questions in the flow diagram). |
| Outcome data | 15* | Table 4 |
| Main results | 16 | (*a*) Table 2, Table 3, Table 4 and S1 Table |
|  |  | (*b*) LogCMTR_IgG3_AMA1 boundary: -.3259806  LogCMTR_IgG3_MSP1 boundary: -.5034789  LogCMTR_IgG3_3D7 boundary: -.5747749  LogCMTR_IgG3_FC27 boundary: -.47684  LogCMTR_IgG3_MSP3 boundary: -.6220508  LogCMTR_IgG3_R0 boundary: -.8644725  LogCMTR_IgG3_R2 boundary: -.6651536 |
|  |  | (*c*) Not applicable |
| Other analyses | 17 | Figure 2 and Table 3 |
| Discussion | | |
| Key results | 18 | Abstract and Discussion paragraph 1 to 5 |
| Limitations | 19 | Abstract and Discussion paragraph 6 |
| Interpretation | 20 | Abstract and Discussion paragraph 6 and 7 |
| Generalisability | 21 | Abstract and Discussion paragraph 7 |
| Other information | | |
| Funding | 22 | Financial Disclosure and Acknowledgments |

*Give information separately for exposed and unexposed groups. **Note:** An Explanation and Elaboration article discusses each checklist item and gives methodological background and published examples of transparent reporting. The STROBE checklist is best used in conjunction with this article (freely available on the Web sites of PLoS Medicine at http://www.plosmedicine.org/, Annals of Internal Medicine at http://www.annals.org/, and Epidemiology at http://www.epidem.com/). Information on the STROBE Initiative is available at http://www.strobe-statement.org.

Flow diagram of the participants


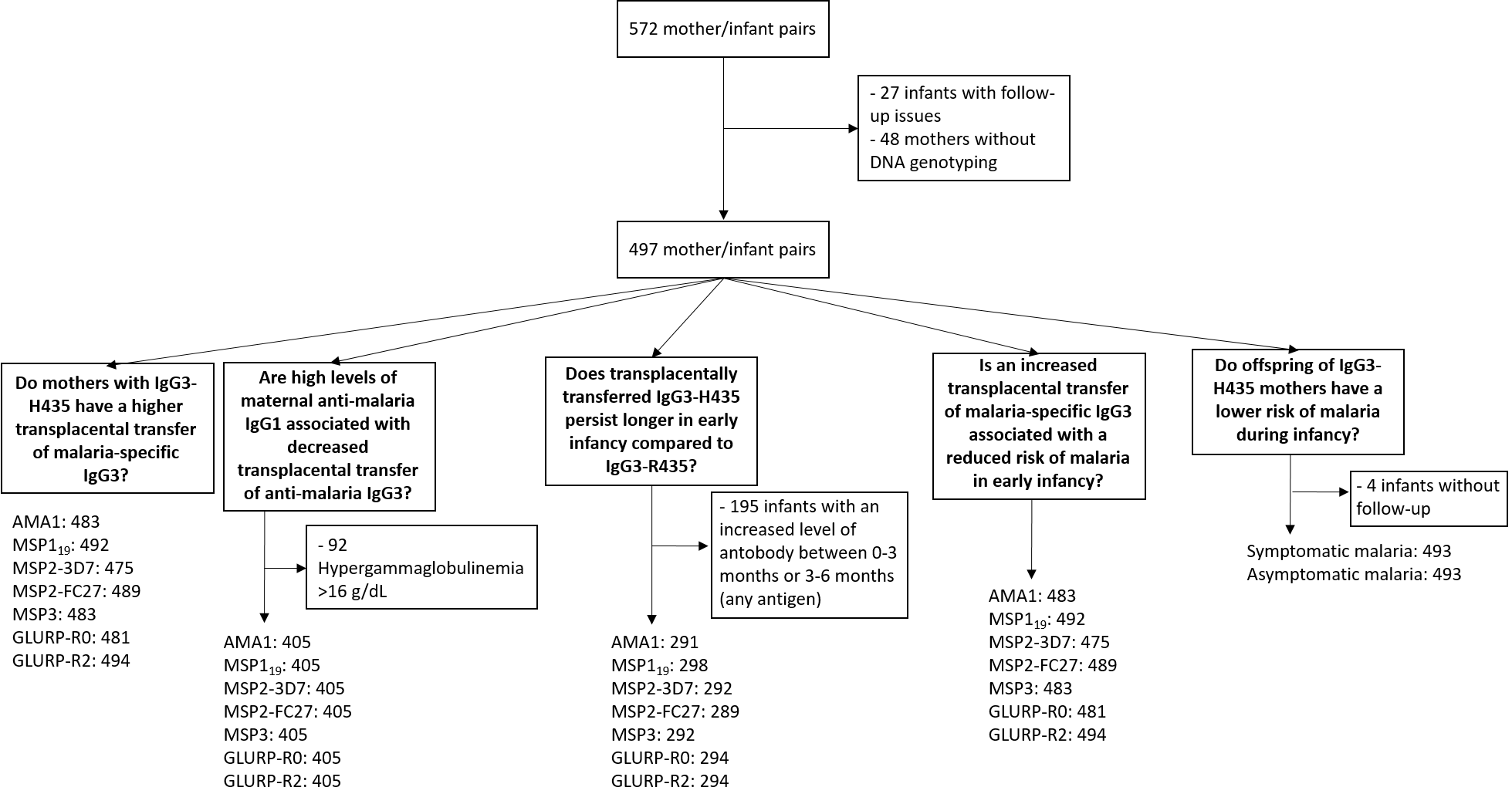

Supplement: S1 Checklist — (DOC) [file pmed.1002403.s001.doc]
