# Supplementary figures and images for: Associations between an IgG3 polymorphism in the binding domain for FcRn, transplacental transfer of malaria-specific IgG3, and protection against Plasmodium falciparum malaria during infancy: A birth cohort study in Benin
Source: PLoS Med. 2017 Oct 9;14(10):e1002403. doi: 10.1371/journal.pmed.1002403 (PMC5633139; doi:10.1371/journal.pmed.1002403)

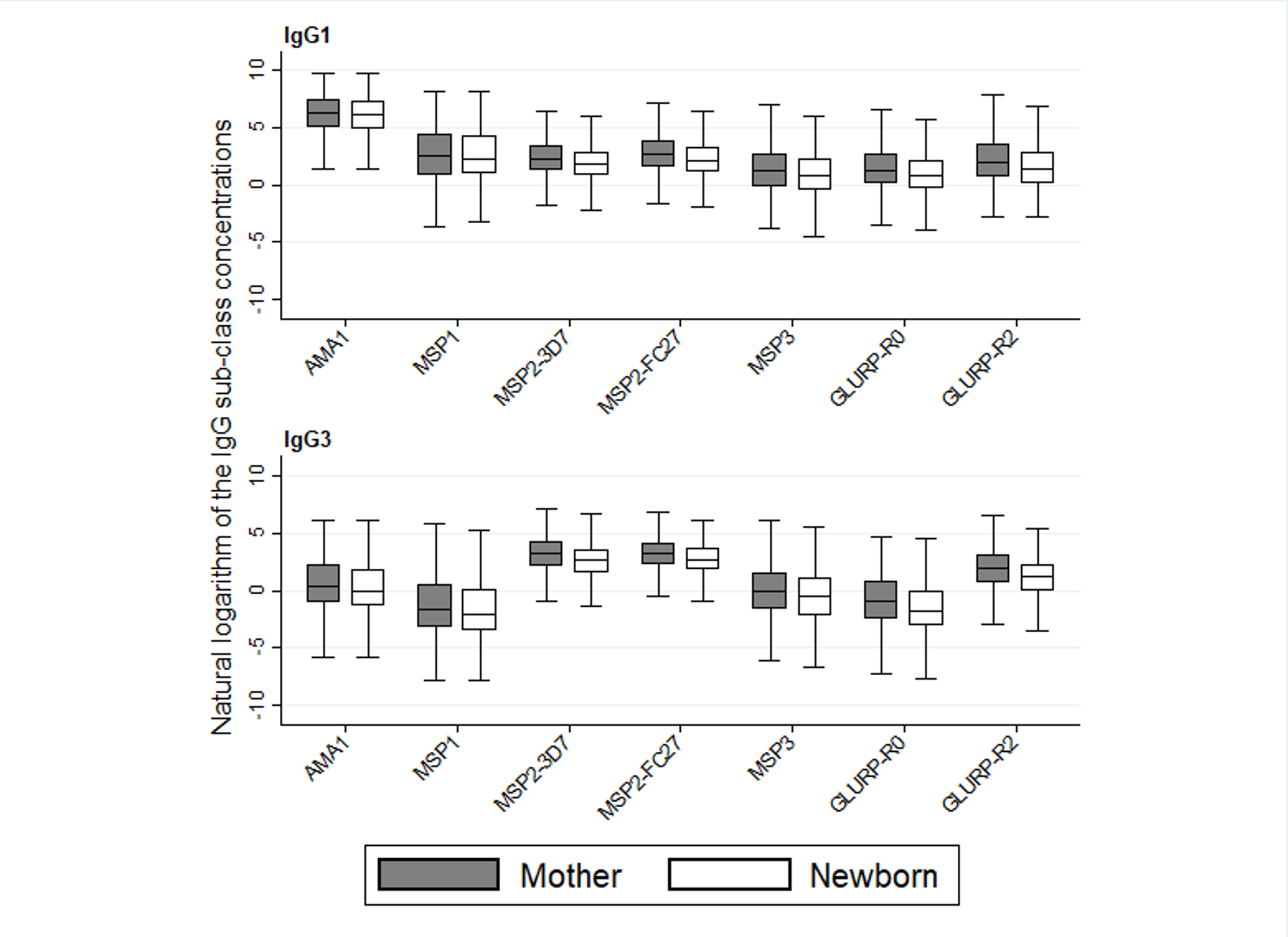

Supplement: S1 Fig — The log-transformed concentrations of IgG1 and IgG3 to malaria antigens are presented in this figure. Maternal levels are in gray and newborn levels are in white. (TIF) [file pmed.1002403.s002.tif]
