# Supplementary material for: Associations between an IgG3 polymorphism in the binding domain for FcRn, transplacental transfer of malaria-specific IgG3, and protection against Plasmodium falciparum malaria during infancy: A birth cohort study in Benin
Source: PLoS Med. 2017 Oct 9;14(10):e1002403. doi: 10.1371/journal.pmed.1002403 (PMC5633139; doi:10.1371/journal.pmed.1002403)
Supplement: S1 Protocol — (DOC) [file pmed.1002403.s003.doc]

# S1. Protocol. A- Description du projet de recherche

# Survenue des premières infections palustres chez le nouveau né : déterminants génétiques, biologiques et environnementaux

## Résumé du projet

Les facteurs conditionnant la survenue des premières infections palustres sont très mal connus alors que le paludisme représente une des premières causes de mortalité dans les pays en développement.

L’importance de l’exposition aux parasites est un des principaux éléments d’un système pathogène faisant intervenir l’hôte, le vecteur et le parasite. L’hétérogénéité de l’espace de vie des jeunes enfants occasionne des variations considérables du niveau de transmission vectorielle, et donc l’étude de la transmission entomologique dans les lieux où vivent les nouveau-nés est indispensable à la compréhension de la dynamique et de l’expression clinique des premières infections. En particulier, dans la situation d’urbanisation croissante de l’Afrique, l’étude du contraste entre zones urbaines et rurales doit permettre de dégager les différences importantes que génèrent ces deux types d’habitats.

Par ailleurs, des facteurs individuels spécifiques de l’hôte jouent également un grand rôle. Par exemple, la notion d’une infection placentaire à la naissance conditionne fortement la probabilité, à niveau égal d’exposition, de développer une infection dans la première année de vie. Les mécanismes biologiques responsables restent à déterminer, notamment l’hypothèse que l'exposition *in utero* à des antigènes du parasite induirait une tolérance immunologique et modifierait la susceptibilité de l'enfant à l'infection et/ou à la maladie. D’autres éléments interviennent dans ce processus de maturation, comme les apports nutritionnels et la rencontre avec d’autres agents infectieux au cours de la petite enfance.

Enfin, le patrimoine génétique de l’hôte joue un rôle primordial, notamment les polymorphismes de gènes impliqués dans le contrôle de l’immunité. Il s’agit d’un contrôle génétique complexe (hétérogénéité génétique), auquel se surajoutent des interactions avec des facteurs individuels (comme l’âge ou la durée d’exposition), environnementaux et comportementaux.

Ces considérations illustrent les interrelations complexes sous-jacentes à la survenue des premières parasitémies palustres. L’ensemble des éléments impliqués, exposition au vecteur, maturation du système immunitaire et facteurs génétiques, doivent donc être pris en compte de manière simultanée et intégrée lors du suivi des individus à risque.

Dans ce programme nous nous proposons de suivre une cohorte de 550 nouveau-nés, entre la naissance et l’âge de 18 mois, dans une zone de forte transmission palustre (sud du Bénin), sur un site rural et un site urbain. Au cours de ce suivi, des données parasitologiques et cliniques seront recueillies. Elles permettront de caractériser les premières infections et les premiers accès cliniques chez les nouveau-nés. Des facteurs de risque individuels (co-infections, statut nutritionnel, variables immunologiques et génétiques) seront mesurés, ainsi que des facteurs environnementaux centrés sur le risque entomologique, qui seront intégrés dans un système d’information géographique.

Nous identifierons les principaux facteurs de risque de survenue des parasitémies palustres sur trois plans : (1) écologique (transmission vectorielle, habitat urbain/rural), (2) biologique (constitution de la réponse immune) et (3) génétique (contrôle de la susceptibilité individuelle à l’infection). Ces informations permettront de mieux comprendre la dynamique d’apparition et d’expression de ces infections et pourront aider à proposer des interventions en santé publique permettant de protéger prioritairement des groupes à risque ou d’éviter la constitution de situations à risque.

Pour assurer la réalisation de ce programme, la collaboration envisagée associe trois équipes de l’IRD (spécialisées dans l’épidémiologie du paludisme et l’épidémiologie génétique, l’entomologie et la nutrition), fortement implantées sur le terrain, deux équipes de l’Inserm et une équipe du CNRS ayant une compétence reconnue dans l’analyse des données (modèles de survie et modèles hiérarchiques d’une part, épidémiologie génétique de l’autre). L’ensemble des études se déroulera en partenariat étroit avec les structures de recherche béninoises.

Malaria is one of the leading causes of death in developing countries. But the circumstances surrounding early infection are poorly understood. One of the most important factors in the complex malaria pathogenic system – which encompasses the host, the vector and the parasite – lies in exposure to parasites. Diversity in young children’s living conditions is directly related to differences in entomological transmission levels. It is thus of the utmost importance to measure the transmission levels affecting areas where newborns live so that we may understand the dynamics of the clinical expression of early infections. In particular, the comparison of urban and rural areas in the increasingly urbanised African continent should lead to a better knowledge of the major differences between these two types of areas.

Furthermore, host-specific individual factors also play an important part. For instance, placental infection at birth (at similar levels of exposure) is strongly related to the probability of being infected in the first year of life. The biological mechanisms responsible for this are yet to be determined and particularly the hypothesis that *in utero* exposure to parasite antigens leads to a form of immunological tolerance modifying the susceptibility of the child to infection and/or disease. Other factors come into play at this level too, such as nutritional status and contact with other pathogens during childhood.

Finally, the genetic make-up of the host is a crucial factor, especially genetic polymorphism related to immune control. This is a complex form of genetic control (genetic heterogeneity) which interacts with individual (age and duration of exposure), environmental and behavioural factors.

All this is a good illustration of the complexity behind the occurrence of initial malaria parasitaemia. The entire spectrum of factors – exposure to infection, maturation of the immune system and genetic factors – must be taken into account simultaneously during a follow-up of individuals at risk.

In this proposal, we wish to study a cohort of 550 newborns from birth to age 18 months in an area of intense transmission (southern Benin) comprising a rural and an urban area. During the study, parasitological and clinical data will be collected. These will allow us to describe early infections and febrile episodes in newborn children. We will also measure individual (co-infections, nutritional status, immunological and genetic parameters) as well as environmental factors focusing on entomological risk, which will be entered into a geographic information system.

We will identify the main factors for malaria parasitaemia which we have grouped into three categories: (1) ecological (vectorborne transmission, rural/urban habitat), (2) biological (the immune response) and (3) genetic (individual control of susceptibility to infection). This information will allow us to better understand the dynamics behind the initial occurrence and expression of these infections and will enable us to suggest public health interventions to protect groups at risk, as well as for avoiding situations liable to pose threats.

This programme brings together three teams from IRD (specialised in the epidemiology of malaria and genetic epidemiology, entomology and nutrition) which are present in the field, two teams from INSERM and one team from CNRS with recognised achievements in the field of data analysis (survival models and hierarchical models as well as genetic epidemiology). The entire research will be led in conjunction with the Beninese scientific institutions.

## Objectifs

Ils se situent à trois niveaux : descriptifs, étiologiques, et interventionnels :

- Identification des principaux facteurs de risque de survenue des parasitémies palustres dans les 18 premiers mois de vie, sur les plans :
  - Ecologique (transmission vectorielle, habitat urbain/rural)
  - Biologique (constitution de la réponse immune)
  - Génétique (contrôle de la susceptibilité individuelle à l’infection)
- Compréhension, dans une approche globale, de la dynamique d’apparition et d’expression de ces infections
- Proposition d’interventions en santé publique permettant d’identifier des populations dans des espaces à risque et de protéger prioritairement des groupes à risque ou d’éviter la constitution de situations à risque

## Contexte scientifique, état de l’art et bibliographie

Le paludisme touche 90 pays de la zone intertropicale et plus de 2 milliards d’individus (36% de la population mondiale) sont soumis au risque infectieux. Bien que variables, les estimations de la morbidité et de la mortalité liées à cette maladie confirment son importance puisqu’on rapporte entre 300 et 500 millions de cas cliniques et de 1 à 3 millions de décès par an. Les jeunes enfants et les femmes enceintes sont les deux populations particulièrement à risque et 80% de la mortalité se concentre sur les jeunes enfants africains (The World Health report, 2002). En effet, dans les zones de stabilité de l’endémie palustre, c’est la période précédant l’acquisition d’une immunité protectrice qui correspond au risque maximal de développer un paludisme grave.

La probabilité de développer une parasitémie ou un accès clinique (bénin la plupart du temps, et grave pour une petite proportion des cas), n’est cependant pas équivalente d’un sujet à l’autre. De plus, cette probabilité varie considérablement dans le temps et dans l’espace. En premier lieu, l’intensité de l’exposition aux parasites (et donc, la fréquence des contacts avec des anophèles infectés) est un élément fondamental. Cette variation de l’exposition à la transmission du paludisme dépend de nombreux facteurs qui concourent à former un système pathogène spécifique au paludisme – un ensemble où interviennent des caractéristiques liées à l’hôte, au vecteur et à l’agent pathogène et au milieu de vie des populations exposées. L’interaction entre les différentes composantes du milieu contribue à modeler une expression spécifique de la maladie pour un espace et des populations donnés. Plus précisément, l’étude de la transmission entomologique dans les lieux où vivent les nouveau-nés, contemporaine de leurs premiers mois de vie, est indispensable à la compréhension de la dynamique et de l’expression clinique des premières infections. Sur le plan écologique, l’hétérogénéité de l’espace de vie des jeunes enfants peut occasionner des variations considérables du niveau de transmission vectorielle (nombre de piqûres par homme et par an). L’environnement de vie est considéré ici dans son acceptation au sens large : l’environnement naturel (biotope, pluviométrie), ou artificiel (présence de collections d’eau permanentes telles que barrage ou irrigation) qui sont autant de facettes de modes d’occupation de l’espace (Koram et al., 2000 ; Ndiaye et al2001). Ces caractéristiques spatiales liées aux variations dans l’intensité de la transmission varient considérablement selon l’échelle à laquelle on les considère : le niveau de transmission vectorielle mesurée à l’échelle d’un village peut connaître des fluctuations importantes entre habitations individuelles.

Il est désormais bien établi que l’intensité de la transmission du paludisme est moindre dans un contexte urbain, même si cette réalité demande à être nuancée en Afrique subsaharienne où le paysage urbain est souvent un mélange de zones bâties et d’espaces végétaux où des dynamiques de transmission du paludisme peuvent s’installer localement. Néanmoins, dans la situation d’urbanisation croissante actuelle de l’Afrique – 37 % de la population en 2000 – l’étude du contraste entre zones urbaines et rurales doit permettre de dégager les différences importantes que génèrent ces deux types d’habitats, tant sur le plan de la stricte exposition aux vecteurs que d’éléments liés aux comportements des populations humaines.

Par ailleurs, des facteurs individuels spécifiques de l’hôte, tels que l’existence d’un premier contact avec le parasite pendant la vie intra-utérine, semblent jouer un grand rôle dans la susceptibilité du nouveau-né aux infections post-natales. Un suivi épidémiologique et parasitologique de nouveau-nés que notre équipe avait effectué au Cameroun a ainsi montré que les enfants nés d'une mère présentant un placenta infecté développaient une première parasitémie et un premier accès palustre plus tôt, après la naissance, que des enfants nés d’un placenta non infecté (Le Hesran *et al.*, 1997). Cette observation vient d’être confirmée par un nouveau suivi de cohorte en Tanzanie (Theonest et al., 2005). Les mécanismes biologiques responsables restent à déterminer, notamment l’hypothèse que l'exposition *in utero* à des antigènes du parasite induirait une tolérance immunologique et modifierait la susceptibilité de l'enfant à l'infection et/ou à la maladie. Quoi qu’il en soit, de nombreux arguments existent pour penser que la compétence à répondre à des antigènes spécifiques de *P. falciparum* s’acquiert très tôt *in utero* (Fievet *et al.*, 1996; King *et al.*, 2002; Ismaili *et al.*, 2003 ; Suguitan *et al.* 2003 ; Xi *et al.*, 2003).

D’autres éléments intervenant dans l’acquisition de l’immunité, peuvent également influencer la réponse aux premières infections palustres. D’une manière générale, l’âge de l’enfant conditionne son comportement dès les premiers mois de vie (apprentissage de la marche, baignades), et influe ainsi sur le risque, quasi-inexistant à la naissance, de contracter d’autres infections parasitaires que le paludisme. Ces co-infections sont fortement suspectées d’influer sur le développement de l’immunité par le biais d’interactions entre parasites. C’est ainsi que notre équipe a pu montrer que l’infection par des helminthes diminuait l’intensité de l’impaludation (Briand *et al.*, 2005 ; Brutus *et al.*, 2006). De même, l’état nutritionnel et le développement du jeune enfant interagissent fortement et ont un fort retentissement sur sa réceptivité aux infections. En particulier, les carences globales, en macronutriments, ou plus spécifiques en certains micronutriments, altèrent le développement et le fonctionnement du système immunitaire, ce qui en retour favorise les infections répétées qui retentissent négativement sur la croissance de l’enfant (Scrimshaw, 1997).

Enfin, le patrimoine génétique de l’hôte joue un rôle primordial et notamment les polymorphismes de gènes impliqués dans le contrôle de la réponse immune (Hill *et al.*, 1992; Garcia *et al.*, 1998a; Rihet *et al.*, 1998a, Flori *et al.*, 2003). Cependant des résultats discordants soulignent la complexité de ces phénomènes. C’est le cas, par exemple, d’associations entre certains polymorphismes du gène codant pour le TNFα et les accès sévères trouvés significatives en Gambie et non au Kenya (Knight *et al*., 1999), ou encore du rôle protecteur de certains haplotypes du gène codant pour l’Interleukine 10 mis en évidence au cours d’enquêtes en population générale et non confirmé sur des données familiales (Wilson *et al.*, 2005). Ces discordances peuvent trouver leur origine dans la réelle complexité de ce contrôle génétique (hétérogénéité génétique). Elles peuvent aussi être liées à la caractérisation des phénotypes, dont le choix influe de manière importante sur les résultats. D’autres éléments peuvent également jouer, tels que l’existence d’infections parasitaires multiples qui vont moduler la survenue des parasitémies palustres, ou d’autres facteurs individuels, environnementaux et/ou comportementaux susceptibles d’interférer avec les facteurs génétiques (Courtin et al., 2006a ; 2006b). C’est ainsi qu’il a été montré pour d’autres pathologies infectieuses tropicales que la non prise en compte des déplacements ou du statut migratoire des sujets pourrait masquer l’effet de facteurs génétiques (Garcia et al., 2002 ; Alcaïs et al., 1997).

Citons enfin, parmi les facteurs individuels qui interagissent avec le patrimoine génétique des sujets, les variables dépendantes du temps, comme l’âge et la durée d’exposition. L’âge est une variable particulièrement complexe puisqu’elle sert à mesurer non seulement le vieillissement des individus mais également la maturation des effecteurs du système immunitaire ou encore la durée d’exposition d’un sujet à l’agent infectieux. Dans ce dernier cas, nous avons montré que la non prise en compte de ce facteur pouvait également masquer l’effet de facteurs de risque génétiques (Courtin et al., 2006a).

Dans le cas du paludisme, nous avons montré qu’il existait une forte interaction entre le contrôle du niveau d’infection par *P. falciparum* et l’âge des sujets dans une population exposée de manière forte à l’infection (Garcia *et al.*, 1998b). Ce résultat indique que l’acquisition de l’immunité avec l’âge n’est pas systématique mais dépend, entre autres, du polymorphisme génétique des individus. La confirmation de ce résultat dans une population génétiquement différente et différemment exposée au risque d’infection confirme que ce phénomène ne dépend pas des conditions environnementales (Rihet *et al.*, 1998b). Cette prémunition, souvent décrite comme un processus progressivement acquis pendant l’enfance au fur et à mesure des infections, pourrait donc ne pas être atteinte par certains individus, certainement plus sensibles à l’infection et qui décèdent dès les premières expositions au parasite (Baird *et al.*, 1995). Une conséquence importante de ces constatations est que l’effet de facteurs génétiques dans le contrôle de l’infection palustre doit être recherché très tôt dans la vie des individus.

Ces considérations illustrent les interrelations complexes sous-jacentes à la survenue des premières parasitémies palustres. C’est ainsi que dans une perspective de description, mais aussi de prédiction, ces éléments, exposition au vecteur, maturation du système immunitaire et facteurs génétiques, doivent être pris en compte de manière simultanée et intégrée lors du suivi des individus à risque.

Les facteurs à l’origine des premières parasitémies et de leur expression clinique sont très mal connus dans les zones de paludisme stable, tout au plus peut-on situer approximativement la période de susceptibilité maximale des individus entre un et cinq ans. Or, une meilleure connaissance de ces éléments est indispensable à la compréhension des mécanismes physiopathologiques responsables de la morbidité et de la mortalité palustres. Elle a également une grande importance en santé publique, dans le sens où elle peut conduire à l’identification de sous-populations davantage exposées aux risques et donc susceptibles d’être protégées en priorité, mais aussi de conjonctions de facteurs (écologiques, collectifs et individuels) que des interventions simples permettraient d’éviter. Notamment, il serait souhaitable de pouvoir caractériser les variations du risque palustre, et la comparaison d’espaces ruraux et urbains dans toute leur complexité peut à cet égard apporter des informations précieuses.

## Méthodes et programmes des travaux

L’ensemble des activités de ce programme repose sur le suivi longitudinal d’une cohorte de nouveau-nés de la naissance jusqu’à l’âge de 18 mois. Cette cohorte sera mise en place au Bénin dans deux zones : Tori Bossito, zone rurale où la transmission du paludisme est permanente et varie selon les précipitations et Ouidah, ville de 35 000 habitants dans laquelle la transmission du paludisme, de type urbaine, est plus faible et hétérogène dans l’espace. Le suivi de cette cohorte s’appuie sur la présence de l’équipe de l’UR010 de l’IRD qui possède une grande expérience de ce travail de terrain. La mise en place récente d’un laboratoire de biologie permettra en outre de réaliser l’ensemble des travaux de parasitologie, d’immunologie et de génétique à Cotonou. Chacune de ces étapes sera réalisée en étroit partenariat avec la Faculté des Sciences de la Santé (FSS) et l’Institut des Sciences Biomédicales Appliquées (ISBA) de Cotonou.

#### 4-1 Détermination du nombre de sujets nécessaires

En considérant comme critère de jugement principal la survenue d’une infection palustre après la naissance, il est difficile de calculer avec précision le nombre de sujets nécessaire par groupe (enfants nés de mères dont le placenta était infecté, vs non infecté), dans la mesure où nous ne connaissons pas l’incidence de l’infection chez les nouveau-nés dans cette zone particulière. Cependant, nous pouvons extrapoler à notre région de travail les résultats d’une enquête que nous avions réalisée au Cameroun, zone de forte transmission (Le Hesran et al., 1997). Ce suivi d’une cohorte de nouveau-nés avait montré un excès d’infections palustres dans la première année de vie de 15% chez les enfants dont le placenta était infecté (65% d’enfants parasitémiques) par rapport à ceux dont le placenta n’était pas infecté (50% d’enfants parasitémiques). L’intensité de la transmission étant moindre dans le sud du Bénin, et le délai de survenue des premières infections probablement plus important qu’au Cameroun, on peut en première approximation estimer les proportions respectives d’infections dans les deux groupes après un an de suivi à 50% (placenta infecté) et 35% (placenta non infecté).

Compte tenu des éléments ci-dessus, pour calculer la taille de l’échantillon nécessaire à la mise en évidence d’une différence du taux d’incidence entre les deux groupes, le calcul peut être basé sur la simple comparaison des pourcentages à un an, soit une différence de 15%. Si l’on suppose un risque de première espèce de 5% et une puissance de 80%, pour un ratio placentas non infectés / placentas infectés de 5, le calcul montre qu’un échantillon comprenant 82 enfants avec placentas infectés et 410 avec placentas non infectés serait nécessaire. Soit, en tenant compte d’un taux de perte de vue à un an de 5%, un échantillon total d’environ 520 enfants (que l’on portera à 550 par sécurité), répartis entre les 5 maternités de la zone sanitaire de Tori Bossito (10 villages) et la maternité Kindji de la ville de Ouidah (deux quartiers). Ce schéma permettra d’obtenir environ 70 à 80 recrutements par mois. En considérant une prévalence de l’infection placentaire de l’ordre de 12%, en 10 mois de recrutement le nombre nécessaire d’enfants nés d’un placenta infecté sera inclus. Cette prévalence d’infection placentaire est estimée à partir d’une enquête réalisée en milieu urbain et péri-urbain en 2005 par notre équipe.

#### 4-2 Recueil des données au recrutement et lors du suivi

##### 4-2-1 Données cliniques, parasitologiques et génétiques

Lors de leur arrivée en salle d'accouchement, l’étude sera expliquée aux femmes, et un accord de participation leur sera demandé (consentement éclairé signé).

A la naissance, les informations cliniques et parasitologiques suivantes seront recueillies:

- Durée de la gestation, poids et taille du nouveau-né, poids et taille de la mère avant l’accouchement
- Antécédents de la femme : nombre d’enfants, nombre de fausses couches et d’enfants mort-nés …
- Existence ou non d’une infection placentaire définie par l’existence de formes asexuées de *P. falciparum* à l’apposition placentaire
- Présence d’une infection parasitaire dans le sang de cordon et mesure du taux d’hémoglobine
- Présence d’une infection parasitaire dans le sang périphérique de la mère et mesure du taux d’hémoglobine

Les nouveau-nés seront suivis 18 mois  :

- Deux fois par semaine, une surveillance active des accès palustres sera réalisée : la température axillaire des enfants sera mesurée lors de passages systématiques au domicile des enfants. En cas de fièvre (température axillaire supérieure ou égale à 37°5), ou d'histoire de fièvre rapportée par la famille depuis la visite précédente, une goutte épaisse (GE) sera réalisée. Un examen clinique sera effectué à la recherche d’arguments en faveur de l’origine non palustre de la fièvre. L’existence d’une prise médicamenteuse (automédication) sera notée. Un accès palustre est défini comme l’association d’une température axillaire supérieure ou égale à 37°5, ou d'histoire de fièvre rapportée par la famille depuis la visite précédente ET d’une parasitémie supérieure à 2500 trophozoïtes par μl (WHO, 2003). Dans cette éventualité, les enfants seront traités conformément aux recommandations du Programme National de Lutte contre le Paludisme.
- Tous les mois, une GE sera effectuée systématiquement afin de mesurer la densité parasitaire asymptomatique de chaque enfant. Un questionnaire alimentaire sera également recueilli au cours de la visite mensuelle. La survenue d’épisodes morbides dans la fratrie des moins de 5 ans depuis la dernière visite sera systématiquement recherchée par interrogatoire lors de cette visite mensuelle. Cette information sera utilisée pour orienter la recherche et l’analyse des interactions entre les facteurs de risque génétiques et environnementaux (*cf. infra* 4-3-5-b)
- Tous les mois jusqu’à 6 mois puis tous les trimestres, l’enfant sera pesé et mesuré. Les mêmes mesures anthropométriques seront recueillies tous les 6 mois dans la fratrie des moins de 5 ans. Les parents seront pesés et mesurés au cours de la première visite à domicile (*cf. infra* 4-2-3-c).
- A 3, 6, 12 et 18 mois, un prélèvement de sang capillaire sera effectué sur EDTA pour les études immunologiques (*cf.infra* 4-2-2) et pour le dosage du taux d’hémoglobine
- Nous recueillerons au cours du suivi des informations sur le portage d’helminthes intestinaux et de bilharziose urinaire. L’existence de co-infections, bien qu’ayant probablement des prévalences très faibles à ces âges, sera prise en compte dans l’analyse et l’interprétation des résultats.
- Les anticorps antisporozoïtes, indicateurs du niveau de transmission vectorielle auquel sont soumis les individus seront dosés pour chaque sujet et ses parents à l’issue du suivi, lors des prélèvements sanguins destinés aux dosages génétiques.

Au cours de ce suivi, les parents de l’enfant feront l'objet d'un prélèvement de 10 ml de sang sur EDTA. Le sang sera centrifugé et le buffy coat (couche leuco-plaquettaire) aliquoté et congelé pour extraction de l’ADN génomique en vue des études génétiques (séquençage et génotypage des gènes candidats ainsi que des polymorphismes du globule rouge).

##### 4-2-2 Données immunologiques

L’étude immunologique a pour objectif d’évaluer le passage transplacentaire d’anticorps anti-*P. falciparum* particuliers et leur évolution chez l’enfant. Elle sera réalisée dans le sang de cordon ainsi qu’à 3, 6, 12 et 18 mois et sera comparée à celle de la mère dans le sang périphérique à l’accouchement. Nous nous intéresserons particulièrement à la réponse IgG et à ses composantes cytophiles (IgG1 et IgG3), qui se trouvent le plus souvent associées à la protection clinique en raison de leur intervention dans le processus de phagocytose des érythrocytes infectés par *P. falciparum* (Groux et Gysin, 1990). La détermination des IgM, qui ne traversent pas la barrière placentaire, permettra de rechercher la présence d’une réponse propre de l’enfant à la stimulation par des antigènes palustres rencontrés *in utero* (Xi et al, 2003) ou après la naissance. L’évolution relative des IgM et des IgG devrait permettre d’évaluer l’acquisition de la réponse immune spécifique de l’enfant. Seule, la composante anticorps de la réponse immune sera abordée dans ce programme, le volet complémentaire et tout aussi important de la réponse à médiation cellulaire étant envisagé dans un autre appel d’offre du Ministère des Affaires Etrangères.

L’antigène PfEMP1 (*P. falciparum* Erythrocyte Membrane Protein 1) de *P. falciparum* retiendra particulièrement notre intérêt pour son rôle dans les phénomènes de séquestration des hématies infectées dans les organes profonds, résultat de la cytoadhérence entre cet antigène exprimé à la surface de l’hématie infectée et des récepteurs situés notamment au niveau de la microcirculation cérébrale ou des espaces intervilleux placentaires (Miller et al., 2002). Cet antigène, qui contribue grandement aux phénomènes morbides de l’infection palustre (comme par exemple le neuropaludisme, ou l’appauvrissement des échanges nutritifs materno-fœtaux) possède la capacité de varier, étant codé par la famille multigénique *var*, constituée de 60 gènes différents par génome (Smith et al., 2001). Un seul antigène variant est exprimé au cours d’un cycle intra-érythrocytaire du parasite, remplacé éventuellement par un autre variant au cours d’un cycle suivant (Sherf et al., 1998). Chacun de ces antigènes appelle une réponse anticorps spécifique, qui contribue pour une large part à la construction avec l’âge d’une immunité naturelle efficace (Marsh et al., 1989 ; Bull et al., 1998).

Les anticorps dirigés contre les antigènes parasitaires variants exprimés à la surface d’hématies infectées par plusieurs isolats plasmodiaux locaux (3) isolés chez des enfants de notre cohorte ainsi que par une souche de référence seront déterminés par la technique de cytométrie en flux. Cette technique permet d’identifier simultanément, par des combinaisons d’émission de fluorescences à différentes longueurs d’ondes, les hématies infectées et la proportion d’entre elles qui sont revêtues d’anticorps spécifiques des antigènes variants de surface exprimés par l’isolat parasitaire étudié (Piper et al., 1999). Cette première appréciation de la réponse anticorps dirigée contre PfEMP1 sera complétée par la détermination par la technique ELISA de la réponse anticorps dirigée contre des protéines recombinantes et/ou des peptides reproduisant des domaines semi-conservés du même antigène (Stalsooe T et al., 1998 ; Lusingu et al., 2006).

A terme, l’étude immunologique permettra de définir pour chaque enfant un profil de réponses anticorps (spécifiques d’antigènes particuliers, décomposées en isotypes), qui sera confronté aux autres données collectées dans le programme.

##### 4-2-3 Données environnementales et comportementales

1. Mesure du risque entomologique

Les informations suivantes seront recueillies sur la cohorte :

- Utilisation d’une moustiquaire de lit, imprégnée ou non,
- Utilisation de répulsifs anti-moustiques, Recours à d’autre moyens de lutte contre les moustiques (aérosols, serpentins, etc)
- Prise de prophylaxie médicamenteuse,
- Distance des habitations aux points d’eau
- Données climatologiques et sur la végétation

Des mesures entomologiques de la transmission vectorielle seront effectuées dans les villages de l’enquête, à divers moments de l’année en fonction de l’intensité et de la fréquence des précipitations.

Une cartographie précise de chaque village sera faite pour identifier les différents gîtes (permanents/temporaires) au cours de l'année et leur localisation par rapport aux habitations. L'évaluation de la transmission sera faite toutes les 6 semaines par les méthodes classiques utilisées en entomologie: captures de nuit, sur sujets humains, à l’intérieur et à l’extérieur des habitations et récoltes de la faune résiduelle matinale, détermination spécifique des spécimens récoltés. Les adultes, non disséqués, seront conservés à –20°C et les niveaux de transmission seront déterminés au laboratoire par ELISA CSP.

1. Intégration des données environnementales dans un SIG

Un des principaux objectifs de la géographie de la santé est d’étudier le rôle pathogène du milieu et les conséquences d’une mauvaise santé sur l’environnement social, matériel, sur les activités et sur la maîtrise de l’espace. Dans le cadre de cette recherche, l’analyse géographique est particulièrement adaptée à la caractérisation et à la mesure des facteurs de l’environnement – entendu ici au sens large du milieu de vie d’une société, c'est-à-dire de l’ensemble des facteurs physiques et humains qui entrent en interaction, formant le support de vie d’une société que cette dernière contribue à modeler. Ces facteurs « environnementaux » sont susceptibles d’être utilisés comme facteurs d’ajustement dans toutes les analyses de cette étude.

Toutes les informations recueillies, particulièrement les données sur l’environnement et le cadre de vie, ont une dimension spatiale – elles peuvent être représentées sur une carte. Nous proposons d’intégrer les informations recueillies pour l’enquête dans une base de données à référence spatiale, plus communément appelée « système d’information géographique » (SIG). Un SIG est un logiciel pour saisir, stocker, vérifier, intégrer, manipuler, analyser et afficher des données positionnées à la surface de la Terre. Ce type d’outil peut être utilisé pour traiter divers types de cartes. Celles-ci peuvent prendre la forme de plusieurs couches différentes où chaque couche contient des données pour un type d’entité particulier. Chacune des entités est liée à une position sur l’image d’une carte et les couches de données sont organisées de façon à en permettre l’étude et l’analyse statistique.

L’intégration des données dans un SIG permettra d’explorer de manière plus approfondie deux objectifs généraux :

- d’une part, caractériser et comprendre le fonctionnement du système pathogène du paludisme et son expression dans la population dans les espaces étudiés. Nous souhaitons particulièrement développer une approche qui permettra de mesurer de manière aussi précise que possible les variations spatiales du risque palustre compte tenu du lieu de vie des jeunes enfants.
- d’autre part définir des territoires et populations à risque pour des interventions éventuelles de santé publique.
- Les données intégrées dans la base d’information que nous proposons de constituer proviendront de l’enquête (voir le point a) et de sources complémentaires :
- données issues de la télédétection : images satellite pour la caractérisation des variations spatiotemporelles de la couverture végétale ainsi que de la nature et de l’extension du bâti et de l’utilisation de l’espace ;
- données de série climatologiques historiques et issues de modélisations disponibles pour la région ;
- relevés de terrains pour recueillir la localisation exacte des objets géographiques compris dans l’analyse (maisons, routes, champs, points d’eau…)

1. Etat nutritionnel des enfants

La mesure de l’état nutritionnel de l’enfant repose principalement sur les indicateurs anthropométriques classiques que sont les indices taille-pour-âge et poids-pour-taille. Ces indices s’expriment en Z-scores par rapport aux courbes de référence NCHS/WHO (WHO 1995) et le seuil de -2 Z-scores définit respectivement le retard de croissance et l’émaciation (ou maigreur).

Les enfants de la cohorte seront mesurés couchés, au millimètre près, sur des toises spécifiques, par deux opérateurs entraînés. Les enfants seront pesés nus sur des pèse-bébés mécaniques Seca d’une portée de 2-16 Kg et d’une précision de 10g.

Les mesures anthropométriques seront effectuées à la naissance, puis tous les mois jusqu’à 6 mois et tous les 3 mois ensuite. A la naissance, en fonction de l’âge gestationnel, on estimera le niveau de retard de croissance intra-utérin par comparaison à la distribution en centiles d’une courbe de référence du poids de naissance pour âge gestationnel spécifique par sexe et par race (Alexander, 1996). Par la suite, les mesures anthropométriques permettront le calcul des indices classiques évoqués plus haut ainsi que le calcul précis des vitesses de croissance. Ces mesures anthropométriques seront également effectuées dans la fratrie de moins de 5 ans.

Les caractéristiques de l’alimentation seront appréciées à partir d’un questionnaire mensuel. Chez le nourrisson, seront principalement recueillies les conditions de mise au sein puis d’allaitement. Chez l’enfant plus grand on relèvera le calendrier d’introduction et les diverses caractéristiques de l’alimentation de complément selon les recommandations internationales (WHO, 1998). A partir de 6 mois et jusqu’à 18 mois l’aspect qualitatif du régime sera évalué par rappel des 24 heures et des indices de diversité alimentaires, dont il a été récemment montré qu’ils étaient liés à la croissance (Arimond, 2004), seront construits.

Les mêmes indices anthropométriques (taille-pour-âge et poids-pour-taille) permettront d’apprécier l’état nutritionnel des enfants de la fratrie de moins de 5 ans. Pour les adultes (mère + père) on utilisera l’indice de masse corporelle (IMC = poids, en kg / carré de la taille, en m).

##### 4-2-4 Considérations éthiques

Le protocole et les objectifs de l’enquête seront exposés de manière détaillée aux parents des nouveau-nés qui devront donner leur consentement éclairé. L’ensemble du protocole et les documents destinés à l’information et au recueil du consentement seront soumis au Comité Consultatif de Déontologie et d’Ethique (CCDE) de l’IRD et au Comité National d’Ethique du Bénin. L’approbation de ces deux institutions sera nécessaire à la mise en œuvre du programme.

#### 4-3 Analyse des données

##### 4-3-1 Stratégie générale

A l’issue du suivi nous disposerons des données suivantes :

- Parasitologiques et cliniques : densité plasmodiale, présence d’hyperthermie, prises médicamenteuses, signes cliniques d’infections non palustres
- Immunologiques : niveau et cinétique d’acquisition des anticorps
- Individuelles : âge, sexe, période de naissance dans l’année, ethnie, indices nutritionnels et alimentaires
- Maternelles : présence d’une infection placentaire, antécédents obstétricaux
- Ecologiques : niveau de transmission vectorielle, distances aux gîtes

Dans un premier temps, nous chercherons à caractériser les facteurs de risque liés à l’apparition des premières parasitémies, en privilégiant l’hypothèse d’une tolérance induite par un contact intra-utérin avec les parasites (objectivé par l’existence d’une infection placentaire). L’ensemble des co-variables recueillies (données environnementales et entomologiques modélisées par le SIG, données anthropométriques et nutritionnelles, marqueurs de l’immunité humorale, événements morbides intercurrents et prises médicamenteuses…) sera pris en compte. Pour ce faire, deux modèles de régression seront développés. L’un, spécifique aux données censurées, portera sur le délai d’apparition de l’événement d’intérêt (parasitémie positive et/ou paludisme clinique), l’autre, spécifique aux données répétées, s’intéressera au niveau de densité parasitaire évalué au cours du suivi.

Dans un deuxième temps, outre la vérification de l’hypothèse initiale, ce dernier modèle permettra de définir des phénotypes ajustés (cliniques, parasitologiques et immunologiques en cas de liaison de la réponse humorale aux infections palustres), dont le contrôle génétique sera ensuite analysé par des études d’association.

##### 4-3-2 Analyse géographique des données

Nous souhaitons caractériser les variations dans l’espace du risque palustre et comparer les zones rurales/urbaines de l’étude. Pour ce faire, nous proposons de :

- Effectuer une cartographie précise de chaque village ou quartier – cette cartographie comprendra l’ensemble des éléments des espaces étudiés (bâti, voirie, couverture végétale…) ;
- Développer un modèle spatial du risque de transmission vectorielle permettant d’assigner un « risque individuel » selon le lieu d’habitation des sujets. Ce modèle sera développé en comparant plusieurs approches statistiques d’interpolation spatiale des données ponctuelles. Nous souhaitons notamment développer un modèle basé sur le krigeage, méthode stochastique d'interpolation spatiale qui prévoit la valeur d'un phénomène naturel en des sites non échantillonnés par une combinaison linéaire sans biais et à variance minimale des observations du phénomène en des sites voisins ;
- Identifier les différents gîtes (permanents/temporaires) au cours de l'année et leur localisation par rapport aux habitations
- Analyser la relation entre la distance aux gîtes et le risque palustre et selon le contexte rural ou urbain – cette étape passe par une identification des éléments du paysage qui sont associés à un niveau de transmission vectorielle théoriquement plus élevé (par exemple, marécages, points d’eau, etc.)
- Comparer ces niveaux théoriques à la parasitémie et aux accès cliniques éventuellement observés chez les habitants de chaque foyer

Nous espérons ainsi pouvoir caractériser de manière précise les variations spatiales du risque palustre et fournir une série de mesures utilisables comme variables écologiques d’ajustement pour les autres analyses du projet. Idéalement, nous aurons aussi identifié des espaces où le risque palustre est sensiblement plus élevé et qui devraient faire l’objet d’une surveillance de santé publique étroite. Cette approche méthodologique de détection de zones à risque pourrait dès lors être utilisée en routine en milieu tropical subsaharien

##### 4-3-3 Etude des facteurs de risque d’apparition des premières parasitémies asymptomatiques et symptomatiques

Dans un premier temps, un modèle de survie semi-paramétrique sera estimé pour étudier l’association entre la survenue de la première parasitémie ou du premier accès palustre et la présence d’un placenta infecté (Cox, 1972). Ce modèle permettra aussi d’identifier des facteurs de risques potentiels à partir des co-variables recueillies à l’entrée et au cours du suivi.

Dans un deuxième temps, la succession d’événements récurrents chez un même individu sera prise en compte, ce qui n’avait pas été effectué dans les études précédentes (Le Hesran *et al*., 1997 ; Mutabingwa *et al*. 2005). Dans ce cadre, la première parasitémie (ou le premier accès palustre) ainsi que les épisodes suivants du même individu seront analysés par des méthodes appropriées basées sur une extension du modèle de Cox (Kelly and Lim, 2000). Cette analyse permettra d’apprécier si les associations identifiées pour le premier épisode, le sont aussi pour les épisodes suivants.

##### 4-3-4 Etude des facteurs de risque de niveau de densité parasitaire asymptomatique

Un modèle linéaire mixte gaussien sera estimé pour étudier les associations entre les densités parasitaires répétées et les co-variables. Ce modèle permet de prendre en compte la dépendance entre les observations, dépendance créée par la répétition des mesures chez le même enfant. A partir du modèle, les prédictions de la valeur moyenne de la densité parasitaire pour chaque sujet pourront être calculées en utilisant les estimateurs bayésiens empiriques. Ces prédictions seront utilisées comme phénotype quantitatif dans l’analyse d’association.

##### 4-3-5 Analyses d’épidémiologie génétique : étude d’association

Les analyses épidémiologiques qui précèdent permettront de définir, pour chaque sujet, les phénotypes d’intérêt suivants : délai d’apparition de la première parasitémie et du premier accès palustre ; niveau de densité parasitaire asymptomatique. Ces phénotypes seront une mesure précise de la réponse de l’hôte aux premières infections palustres au cours des 18 premiers mois de vie.

L’identification d’éventuelles associations entre les phénotypes d’intérêt et des polymorphismes de gènes candidats ne sera qu’une première étape de ce projet. En effet, des développements ultérieurs prévoient des études de l’expression des polymorphismes identifiés. La présente demande de financement ne concerne pas ces extensions du programme.

Les résultats de l’analyse d’association seront confrontées aux résultats des réponses anticorps obtenues pour l’enfant, afin d’appréhender le rôle du polymorphisme des gènes de cytokines (*cf.* ci-dessous) sur le processus de maturation du système immunitaire.

- - - 1. Exploration des gènes candidats

Préalablement aux études d’épidémiologie génétique, l’ensemble des analyses de génétique moléculaire (extraction de l’ADN ; séquençage des gènes candidats en vue de l’identification des polymorphismes d’intérêt ; génotypage de ces polymorphismes) sera effectué à Cotonou et à Paris.

Les gènes candidats seront déterminés en fonction des résultats d’études antérieures menées par le groupe au Cameroun et au Sénégal, ainsi que sur la base de la littérature.

Des analyses familiales de ségrégation ont révélé l’existence d’un contrôle génétique complexe de la réponse à l’infection palustre, qu’il s’agisse du niveau de densité parasitaire (Garcia *et al.*, 1998a, Rihet *et al.* 1998a) ou des réponses IgG et de sous-classes d’IgG (Stirnadel et al., 2000). Plusieurs régions chromosomiques, contenant de nombreux gènes de cytokines ou de récepteurs de cytokines ont été pressenties pour leur rôle dans ce contrôle. Au Cameroun, l’équipe responsable du projet a mis en évidence l’intérêt de la région q31-q33 du chromosome 5 de l’homme. Cette région contient un cluster de gènes impliqués dans le contrôle de la réponse immune à différents pathogènes comme le gène codant pour l’Interleukine (IL)-3, l’IL-4, l’IL-5, l’IL-13, le Colony Stimulator Factor-2 (CSF-2) et le Immune Regulatory Factor- (IRF-1). Certains de ces gènes codent pour des cytokines sécrétées par les lymphocytes Th2, inductrices de la production d’anticorps par les lymphocytes B (IL-4, IL-5, IL-13). Il est probable que le polymorphisme de ces gènes conduise à des protéines différentes sur le plan fonctionnel, comme cela a été établi entre le polymorphisme du gène codant pour l’IL-4 et la production d’IgG en réponse à plusieurs antigènes des stades sanguins asexués de *P. falciparum* (Luoni et al., 2001). Cette région contient également le gène codant pour l’IL-9 et, dans sa partie distale, le gène codant pour l’IL-12. Enfin, cette région et a été identifiée comme impliquée dans le contrôle de l’hyper réactivité bronchique et le niveau d’Immunoglobuline E, dont le rôle dans la protection et la physiopathologie du paludisme est fortement suspecté (Bereczky et al., 2004 ; Perlmann et al., 2000). D’autres gènes candidats seront également inclus dans l’analyse comme le gène codant le TNFα dans la région chromosomique 6p21-p23, dont un polymorphisme a été trouvé associé à l’accès palustre simple (Flori et al., 2003) ou encore les gènes codant pour l’IL-6 et l’IL-10 (Eskdale et al., 1998, Linker-Israeli et al., 1999 ; Courtin et al., 2006 ; sous presse).

Enfin, une étude d’association sur l’ensemble du génome (6000 polymorphismes ; plate-forme Illumina Golden Gate) est actuellement en cours, au Centre National du Génotypage, sur les données provenant d’une cohorte d’enfants sénégalais suivis 3 ans par l’équipe proposante. La présente étude devrait permettre de confirmer et d’affiner certains résultats et d’identifier potentiellement d’autres gènes candidats.

- - - 1. Tests d’association et recherche d’interactions gène gène, gène environnement sur ces gènes candidats

Pour déterminer si les gènes candidats sélectionnés sont impliqués dans les phénotypes d'intérêt, une première étape consistera à déterminer quels seront les marqueurs à tester dans ces gènes après séquençage. En effet, on dispose aujourd'hui de très nombreux marqueurs de type Single Nucleotide Polymorphisms (SNPs) dans les gènes et il est impossible et souvent inutile de les tester tous car ils apportent souvent une information redondante. Nous avons développé une méthode efficace pour sélectionner, dans un gène candidat, la combinaison de marqueurs qui maximisera la puissance de détection (Génin et al., 2001 ; Cousin et al., 2003, 2006). La seconde étape est celle du choix de la méthode d'analyse.

Le protocole de l’étude se prête parfaitement à la mise en place d’une étude d’association familiale basée sur des trios (enfant et parents). La méthodologie proposée est une analyse d’association permettant de comparer la fréquence d’un allèle particulier d’un marqueur génétique entre une population de sujets atteints (porteurs du phénotype) et une population de témoins. Un type particulier d’analyse d’association, appelé TDT, pour Test de Déséquilibre de Transmission (Spielman *et al*., 1993), sera utilisé dans notre analyse. Les familles où au moins un des deux parents est manquant seront analysées par les méthodes comme le TDT en fratrie, ou S-TDT, et le RC-TDT permettant la reconstruction des génotypes parentaux. Les analyses seront réalisées avec les programmes FBAT et PBAT qui permettent la prise en compte de covariables (notamment l’infection placentaire) et de leurs éventuelles interactions avec les facteurs génétiques. Des analyses basées sur une approche régressive conditionnelle pourront également être utilisées. Dans ce cas, des odds ratios conditionnels aux génotypes des sujets pour le polymorphisme considéré sont obtenus.

En plus des analyses individuelles pour chaque SNP, nous envisageons de tester conjointement l'effet des différents marqueurs situés dans un gène (analyse des haplotypes) mais également situés dans des gènes susceptibles d'interagir comme IL-4-IL-13 et leur récepteur IL-4R. En effet, de nombreuses interactions peuvent exister entre variants dans un gène ou dans des gènes différents et, ne pas prendre en compte ces interactions, peut considérablement réduire la puissance de détection des effets de ces variants (Franckel & Schork, 1996). Nous avons développé une méthode permettant de tester conjointement les effets de plusieurs SNPs en considérant simultanément toutes les combinaisons de marqueurs et en évaluant la signification des tests en tenant compte de leur dépendance (Jannot et al., 2003).

Enfin, de la même manière il peut être nécessaire de tenir compte de certains facteurs environnementaux (fréquence, effet propre) pour mettre en évidence les facteurs génétiques(Selinger-Leneman et al., 2003). Nous avons développé une nouvelle méthode qui permet, à partir de données familiales sur la maladie (risque de récurrence chez les germains) et du statut d'exposition du proposant, de déterminer si le facteur environnemental considéré est susceptible ou non d'interagir avec des facteurs génétiques (non connus). L'utilisation de cette méthode sera possible ici grâce aux informations familiales recueillies qui permettront d'avoir une idée de l'état de santé de la famille (antécédents obstétricaux de la mère, état de santé des frères et sœurs du nouveau-né inclus, qui sera évalué par le questionnaire mensuel et les mesures anthropométriques). Une variable sera tout particulièrement prise en compte : il s’agit de l’infection placentaire. En effet la prise en compte de cette variable, et de ses éventuelles interactions avec les polymorphismes testés, pourrait aider à mieux comprendre ce phénomène majeur impliqué dans le contrôle de la réponse de l’hôte aux premières infections palustres et dans le développement de l’immunité spécifique.

La méthodologie présentée ci-dessus entraîne la répétition des tests statistiques. Si le risque d'erreur (par exemple de première espèce) est parfaitement contrôlé lorsque l'on considère un unique test, la question est plus délicate dans le cadre des tests multiples. Une approche classique à ce problème consiste à contrôler le risque global de première espèce (FWER) par des corrections de type Bonferroni ou par le biais de permutations. Parallèlement à ces approches, des méthodes de contrôle du False Discovery Rate (FDR) ont été récemment introduite (Benjamini et Hochberg, 1995). Le principe est de choisir un seuil de significativité de façon à obtenir parmi les observations significatives un taux donné de fausses découvertes. L'avantage d'une telle notion est bien évidemment sa facilité d'interprétation. Plus récemment encore (Efron et al, 2001), cette notion de FDR a été affinée et permet pour chaque test effectué d'estimer la probabilité d’aboutir à une fausse découverte (c'est le local FDR).

D’autres problèmes méthodologiques se posent concernant notamment le nombre de statistiques concurrentes existant pour mesurer l'association génétique à la maladie. En l'absence de critère objectif, le choix de l'une ou l'autre de ces approches est difficile même si le principe reste de retenir celle(s) qui (à niveau égal) maximise(nt) la puissance. Dans le cas de ce projet, nous nous proposons d’optimiser la mesure d’associations génétiques, en comparant de manière exhaustive l'ensemble des statistiques permettant cette mesure d'association.

## Coopération entre équipes, coordination et gestion du projet ; actions de formation

Ce projet développe un étroit partenariat Nord-Sud et Nord-Nord. Ce programme repose sur un travail de terrain lourd et complexe qui devra permettre de disposer, à l’issue du suivi, de données cliniques, biologiques et environnementales de qualité. Ce travail de collecte des données sera coordonné par l’UR010, responsable du programme, en étroite collaboration avec les structures de santé locales et le Programme National de Lutte contre le paludisme (PNLP). Le PNLP du Bénin est engagé dans une importante refonte de sa stratégie de lutte contre le paludisme (distribution de moustiquaires imprégnées, modification de la prise en charge du paludisme de la femme enceinte et de l’enfant …). Cette refonte est fortement accompagnée par les bailleurs de fonds internationaux qui financent une grande part de ce travail. L’IRD (et particulièrement les UR 010 et 016) est associé à cette réforme notamment par le biais de programmes de recherches d’ores et déjà effectués ou en cours (financements IMEA –Institut de Médecine et d’Epidémiologie Appliquée- et MAE –Ministère des Affaires Etrangères-) dont les résultats seront restitués afin d’aider à orienter les actions de lutte du PNLP béninois. Dans le cadre de cette coopération étroite le PNLP nous a aidés à identifier les maternités dans lesquelles le programme sera mené, a participé aux missions de sensibilisation préalables auprès des médecins chefs de zones sanitaires et sera associé à l’ensemble des actions de terrain. Ces travaux, ainsi que le présent projet, intéressent également les pays de la sous région engagés dans des modifications de leur PNLP.

Sur le plan des analyses biologiques le laboratoire de l’UR010 est implanté au sein de la Faculté des Sciences de la Santé (FSS) et de l’Institut des Sciences Biomédicales Appliquées (ISBA) de Cotonou. Ce laboratoire a été équipé fin 2005 sur des fonds propres de l’IRD et sur un financement du MAE et il dispose d’un équipement de pointe dans les domaines de la génétique moléculaire (thermocycleurs, spectrophotomètre pour ADN/ARN, appareil de PCR en temps réel) et de l’immunologie (cytomètre en flux, chaîne ELISA …).

Le programme associera trois Unités de Recherche de l’IRD (UR016 : entomologie ; UR106 : nutrition ; et UR010 : épidémiologie génétique, immunologie et géographie de la santé), deux Unités INSERM (U535 : génétique épidémiologique et structure des populations humaines ; U780 : Recherche en épidémiologie et biostatistique) et une Unité Mixte de Recherche CNRS (UMR CNRS 8071 : Statistique et génome). Les deux unités INSERM ainsi que l’UMR CNRS associées à ce programme travaillent dans le domaine du traitement biostatistique des données ou de l’épidémiologie génétique des maladies multifactorielles.

L'unité INSERM 535 est spécialisée dans l'étude des facteurs génétiques de risque aux maladies complexes. Elle a développé des méthodes d'analyse statistique spécifiquement adaptées à la détection de l'effet d'un gène candidat et à la modélisation de son rôle: identification des polymorphismes les plus probablement impliqués, estimation des risques associés, prise en compte des interactions entre variants génétiques et avec des facteurs environnementaux. Ces développements méthodologiques sont appliqués en collaboration avec des équipes de génétique clinique et moléculaire principalement sur des maladies auto-immunes (polyarthrite rhumatoïde, diabète de type 1, Sclérose en Plaques). Dans ces travaux l'U535 s'est beaucoup intéressée au cluster IL4-IL13 sur le chromosome 5q31-33 et aux interactions avec le gène IL4R (codant pour le récepteur de ces interleukines). Ces gènes sont les principaux candidats de la présente étude.

L’équipe biostatistique de l’U780 apportera sa compétence dans la modélisation des données de durée censurée ou longitudinales. Elle est impliquée de longue date dans le développement des méthodes d’analyses de la survie qui s’étendent notamment à l’analyse d’événements récurrents. Son expertise dans le domaine de la méthodologie des études longitudinales est l’un des éléments sur lesquels repose une collaboration étroite établie avec l’UR010 depuis 2002. Cette collaboration a par exemple permis de mener à bien l’analyse de différentes enquêtes réalisées sur les pathologies parasitaires. Dans le cadre de ce projet, l’équipe apportera aussi ses connaissances dans l’analyse des données corrélées à visée prédictive, problématique déjà abordée au sein d’un suivi de cohorte d’enfants plus âgés au Sénégal.

L’UMR CNRS présente une grande expertise dans le domaine des statistiques en s'intéressant à trois aspects essentiels: les tests multiples, les études de puissances et la recherche d'hétérogénéités cachées. Le laboratoire s'est initialement intéressé à cette problématique dans le cadre des études d'association cas-témoins genome-wide et participe au développement d'un logiciel dédié à l'estimation du local FDR. L'expérience de cette équipe sera un atout pour prendre en compte cette problématique.

L'équipe du coordonnateur a contribué de manière prépondérante depuis plusieurs années (au Cameroun et au Sénégal) à l’étude du contrôle génétique de l’infection par *P. falciparum* ainsi qu’à une meilleure connaissance de l’immunologie du paludisme chez la femme enceinte et le nouveau-né. En outre, cette équipe est parfaitement rodée à la réalisation d'études de cohortes, y compris chez des nouveau-nés recrutés à la naissance. L’association de l’UR 016 et des compétences en géographie de la santé de l’UR 010 permettra une mesure précise des facteurs de risques environnementaux qui englobera non seulement les aspects purement vectoriels mais aussi une analyse précise du biotope et des relations qu’entretiennent les populations à risque avec celui-ci. Enfin, la prise en compte du facteur nutritionnel, tout à fait nouvelle dans cette problématique, permettra de disposer d’une information essentielle dans l’analyse de la maturation du système immunitaire.

Dans ce contexte, le programme permettra la formation académique d’étudiants en biologie, en statistique et en épidémiologie. Le partenariat Nord-Sud facilitera la formation en co-tutelle d’étudiants du Sud dans les différentes disciplines. Parallèlement, des formations appliquées et non académiques seront mises en place permettant également la formation d’ingénieurs et de techniciens d’ores et déjà statutaires (FSS, ISBA ou PNLP) à des techniques de pointe. Cette demande est fortement exprimée par les partenaires du Sud.

## Gestion et pilotage du projet

Les coordinateurs de chacune des équipes participantes (7 au total) composeront le comité de pilotage du projet, qui accueillera également un membre de la FSS, de l’ISBA et du PNLP. Ce comité, dirigé par le coordinateur du projet, se réunira une fois par an. La première réunion se déroulera en Janvier 2007 au cours du congrès de l’Association des Epidémiologistes de Langue Française (ADELF) qui se tiendra à Cotonou.

## Modalités de valorisation

La diffusion des résultats se fera également en étroite collaboration avec le Ministère de la Santé du Bénin ainsi que les structures associées de lutte contre le paludisme aux niveaux national, régional et local. Les résultats seront diffusés en utilisant l'ensemble des voies disponibles afin de s'assurer que les informations pourront profiter tant aux spécialistes qu'aux non-spécialistes. Les moyens utilisés comprendront: 1/ une interaction directe avec les représentants du Ministère de la Santé, 2/ des publications dans des revues scientifiques/médicales; 3/ des réunions de restitution régulièrement organisées en collaboration avec le Ministère de la santé et qui s’adresseront aux médecins et professionnels de la santé à un niveau périphérique ainsi qu’aux populations, 4/ des présentations régulières à l’ensemble des partenaires du PNLP à l’occasion des réunions mensuelles.

## Présentation des équipes partenaires

Ce projet développe un étroit partenariat Nord-Sud et Nord-Nord. Voir paragraphe 6 ; le CV de chacun des membres participant à ce programme est présenté en annexe.

## Autres programmes dont européens

Un programme est actuellement réalisé sur financement du Ministère des Affaires Etrangères français par l’UR10 et l’UR16 dans la ville de Ouidah. Ce programme élaboré et mené en étroit partenariat avec le PNLP s’intéresse à la prévention du paludisme de la femme enceinte et se déroule pour partie dans la maternité de Kindji où devrait se dérouler le présent programme.

# B- Annexe complémentaire

### Références citées dans le projet

Alcais, A., Abel, L., David, C., Torrez, M.E., Flandre, P. and Dedet, J.P., **1997**. Evidence for a major gene controlling susceptibility to tegumentary leishmaniasis in a recently exposed Bolivian population. Am. J. Hum. Genet. 61, 968-979.

Alexander GR, Himes JH, Kaufman RB, Mor J, Kogan M. A United States national reference for fetal growth. Obstet Gynecol*,* **1996** 87:163-8.

Arimond M, Ruel MT (2004). Dietary diversity is associated with child nutritional status: evidence from 11 demographic and health surveys, J Nutr, **2004**, 134:2579-85.

Baird JK. Host age as a determinant of naturally acquired immunity to *Plasmodium falciparum*. Parasitol Today, **1995**, 11:105-11.

Benjamini Y, Hochberg Y Controlling the false discovery rate: a practical and powerful approach to multiple testing J R Stat Soc **1995**. 57:289-300

Bereczky S, Montgomery SM, Troye-Blomberg M, Rooth I, Shaw MA, Farnert A. Elevated anti-malarial IgE in asymptomatic individuals is associated with reduced risk for subsequent clinical malaria. 17Int J Parasitol. **2004** Jul;34(8):935-42.

Briand V, Watier L, Le Hesran JY, Garcia A, Cot M. Co-infection with *Plasmodium falciparum* and *Schistosoma haematobium*: protective effect of schistosomiasis on malaria in Senegalese children? *Am J Trop Med Hyg*  **2005**, 72 (6): 702-707

Brutus L., Watier, L., Briand V., Hanitrasoamampionona V., razanatsoarilala H., Cot M. Parasitic co-infections : does *Ascaris lumbricoides* protect against *Plasmodium falciparum* infection? *Am J Trop Med Hyg*, **2006** (sous presse).

Bull PC, Lowe BS, Kortok M, Molyneux CS, Newbold CI, Marsh K. Parasite antigens on the infected red cell surface are targets for naturally acquired immunity to malaria. Nat Med. **1998** Mar;4(3):358-60.

Courtin D., J. Milet, V. Jamonneau, C. Sese Yeminanga, V. Kande Betu Kumeso, C. Miaka Mia Bilengue, C. Bétard, A Garcia. Association between human African trypanosomiasis and the *IL6* gene in a Congolese population (*Infection Genetics and Evolution* **2006a**, in press)

Courtin, D, L. Argiro, V. Jamonneau, B. Sane, P. N’Guessan, L. N’dri, R. Sanon, A. Dessein, L. Abel, C. Laveissière & A. Garcia. Interest of tumor necrosis factor-alpha -308 G/A and interleukin-10 -592 C/A polymorphisms in human African trypanosomiasis(*Infection Genetics and Evolution* **2006b**, in press)

Cousin E., Deleuze J.F., and Genin E., Selection of SNP subsets for association studies in candidate genes: comparison of the power of different strategies to detect single disease susceptibility locus effects. BMC Genet, **2006**. 7(1): p. 20.

Cousin E., Genin E., Mace S., Ricard S., Chansac C., del Zompo M., and Deleuze J.F., *Association studies in candidate genes: strategies to select SNPs to be tested.* Hum Hered, **2003**. 56(4): p. 151-9.

Cox DR. 1972. Regression models and life tables (with discussion). J Roy Stat Soc, Series B34 :187-220

Efron, B., et al. Empirical Bayes analysis of a microarray experiment. J. Am. Stat. Assoc., **2001**: 96, 1151­1160

Fievet N, Ringwald P, Bickii J, Dubois B, Maubert B, Le Hesran JY, Cot M, Deloron P. Malaria cellular immune responses in neonates from Cameroon. Parasite Immunol, **1996** 18:483-90.

Flori L, Sawadogo S, Esnault C, Delahaye NF, Fumoux F, Rihet P. Linkage of mild malaria to the major histocompatibility complex in families living in Burkina faso. Hum Mol Genet **2003**, 12:375-8

Frankel & Schork Who's afraid of epistasis? Nature Genetics, **1996**, 14: 371-373).

Garcia A, Cot M, Chippaux JP, Ranque S, Feingold J, Demenais F, Abel L. Genetic control of blood infection levels in human malaria: evidence for a complex genetic model. Am J Trop Med Hyg, **1998a**, 58:480-8.

Garcia A, Marquet S, Bucheton B, Hillaire D, Cot M, Fievet N, Dessein AJ, Abel L. Linkage analysis of blood *Plasmodium falciparum* levels: interest of the 5q31-q33 chromosome region. Am J Trop Med Hyg **1998b**, 58:705-9.

Garcia A., Jamonneau V., Sane B., Fournet F., N’Guessan P., N’Dri L., Sanon R., Kaba D., Laveissière C. Host age and time of exposure in Trypanosoma brucei gambiense Human African Trypanosomiasis. *Trop. Med. Int. Health*. **2002**; 7: 429-434.

Genin E., *Selection of single nucleotide polymorphisms for association studies in candidate genes.* Genet Epidemiol, **2001**. 21 Suppl 1: p. S614-9.)

Griffiths MJ Shafi MJ, Popper SJ, Hemingway CA, Kortok MM, Wathen A, Rockett KA, Mott R, Levin M, Newton CR, Marsh K, Relman DA, Kwiatkowski DP. Genomewide analysis of the host response to malaria in Kenyan children. J Inf Dis **2005**, 191:1599-611.

Groux H, Gysin J. Opsonization as an effector mechanism in human protection against asexual blood stages of Plasmodium falciparum: functional role of IgG subclasses.Res Immunol. **1990** Jul-Aug;141(6):529-42.

Hill AV, Elvin J, Willis AC, Aidoo M, Allsopp CEM, Gotch FM, Gao XM, Takiguchi M, Greenwood BM, Townsend ARM, McMichael AJ, Whittle HC. Molecular analysis of the association of HLA-B53 and resistance to severe malaria. Nature **1992**, 360:434-9., 6:123-129

Ismaili J, van der Sande M, Holland MJ, Sambou I, Keita S, Allsopp C, Ota MO, McAdam KP, Pinder M. *Plasmodium falciparum* infection of the placenta affects newborn immune responses. Clin Exp Immunol **2003**;133:414-21.

Jannot AS, Essioux L, Reese MG, Clerget-Darpoux F (2003) Improved use of SNP information to detect the role of genes. Genet Epidemiol 25:158-167)

 Kelly P. J. and LIM L-Y. “Survival analysis for recurrent event data: an application to childhood infectious diseases”, Statistics in Medicine, **2000**, 19, 13-33

King CL, Malhotra I, Wamachi A, Kioko J, Mungai P, Wahab SA, Koech D, Zimmerman P, Ouma J, Kazura JW. Acquired immune responses to *Plasmodium falciparum* merozoite surface protein-1 in the human fetus. J Immunol, **2002**, 168:356-64.

Knight, J.C., Udalova, I., Hill, A.V., Greenwood, B.M., Peshu, N., Marsh, K. and Kwiatkowski, D. A polymorphism that affects OCT-1 binding to the TNF promoter region is associated with severe malaria. Nat. Genet. **1999**, 22, 145-150.

Koram, A.K., Osuwu-Agyei, S., Utz, G., Binka, F.N., Baird, J.K., Hoffman, S.L., Nkrumah, F.K. Severe anaemia in young children after high and low malaria transmission seasons in the Kassena-nankana district of Northern Ghana. Am. J. Trop. Med. Hyg. 2000. 62, 670-674.

Le Hesran J.Y., Cot M., Personne P., Fievet N., Dubois B., Beyemé M., Boudin C., Deloron P. Maternal Placental Infection with *Plasmodium falciparum* and Malaria Morbidity During the First Two Years of Life. Am J. Epidemiol,. **1997**, 146:826-31.

LuoniG., Verra F., Arca B., Sirima BS., Troye-Blomberg M., Coluzzi M., Kwiatowski D., Modiano D., Antimalarial antibody levels and IL4 polymorphism in the Fulani of West Africa. Genes Immun., **2001**. 2:411-414

Lusingu JP, Jensen AT, Vestergaard LS, Minja DT, Dalgaard MB, Gesase S, Mmbando BP, Kitua AY, Lemnge MM, Cavanagh D, Hviid L, Theander TG. Levels of plasma immunoglobulin G with specificity against the cysteine-rich interdomain regions of a semiconserved Plasmodium falciparum erythrocyte membrane protein 1, VAR4, predict protection against malarial anemia and febrile episodes. Infect Immun. **2006** May;74(5):2867-75.

Marsh K, Otoo L, Hayes RJ, Carson DC, Greenwood BM. Antibodies to blood stage antigens of Plasmodium falciparum in rural Gambians and their relation to protection against infection. Trans R Soc Trop Med Hyg. **1989** May-Jun;83(3):293-303.

Miller LH, Baruch DI, Marsh K, Doumbo OK. The pathogenic basis of malaria. Nature, **2002**, 415:673-9.

Ndiaye, O., Le Hesran, J-Y., Etard, J-F., Diallo, A., Simondon, F., Ward, M.N., Robert, V. Variation climatique et mortalité attribuée au paludisme dans la zone de Niakhar, Sénégal, de 1984 à 1996. Cahier d’Etudes et de Recherche francophones, **2001**, 11, 25-33.

Perlmann P, Perlmann H, Looareesuwan S, Krudsood S, Kano S, Matsumoto Y, Brittenham G, Troye-Blomberg M, Aikawa M. Contrasting functions of IgG and IgE antimalarial antibodies in uncomplicated and severe Plasmodium falciparum malaria. Am J Trop Med Hyg. **2000** Mar;62(3):373-7.

Piper KP, Roberts DJ, Day KP. Plasmodium falciparum: analysis of the antibody specificity to the surface of the trophozoite-infected erythrocyte. Exp Parasitol. **1999** Feb;91(2):161-9. practical and powerful approach to multiple testing J R Stat Soc

Rihet P, Abel L, Traore Y, Traore-Leroux T, Aucan C, Fumoux F. Human malaria: segregation analysis of blood infection levels in a suburban area and a rural area in Burkina Faso. Genet Epidemiol **1998a**, 15:435-50.

Rihet P, Traore Y, Abel L, Aucan C, Traore-Leroux T, Fumoux F. Malaria in humans: *Plasmodium falciparum* blood infection levels are linked to chromosome 5q31-q33. Am J Hum Genet **1998b**, 63:498-505.

Schaid DJ. Case-parents design for gene-environment interaction. *Genet Epidemiol* **1999**, 16, 261-273

Scrimshaw NS, SanGiovanni JP. Synergism of nutrition, infection, and immunity: An overview. Am J Clin Nutr*,* **1997***,* 66:464S-77S.

Selinger-Leneman H., Genin E., Norris J.M., and Khlat M., Does accounting for gene-environment (GxE) interaction increase the power to detect the effect of a gene in a multifactorial disease? Genet Epidemiol, **2003**. 24(3): p. 200-7.)

Sherf A, Hernandez-Rivas R, Buffet P, Bottius E, Benatar C, Pouvelle B, Gysin J, Lanzer M. Antigenic variation in malaria : in situ switching, relaxed and mutually exclusive transcription of var genes during intra-erythrpcytic development in Plasmodium falciparum. EMBO J **1998**; 17:5418-26.

Smith JD, Gamain B, Baruch DI, Kyes S. Decoding the langague of var genes and Plasmodium falciparum sequestration. Trends Parasitol **2001**; 17:538-45.

Spielman RC, McGinnis RE Ewens WJ. Transmission test for linkage desequilibrium : the insulin gene and insulin-dependent diabetes mellitus (IDDM). Am J Hum Genet **1993**, 52:506-16.

Staalsoe T, Khalil EA, Elhassan IM, Zijlstra EE, Elhassan AM, Giha HA, Theander TG, Jakobsen PH. Antibody reactivity to conserved linear epitopes of Plasmodium falciparum erythrocyte membrane protein 1 (PfEMP1). Immunol Lett. **1998** Feb;60(2-3):121-6.

Stirnadel HA., Beck HP., Alpers MP., Smith TA. Heritability and segregation analysis of immune response to specific malaria antigens in Papua New Guinea. Genet. Epidemiol. **1999**, 17:16-34

Suguitan AL, Leke RGF, Zhou G F A, Thuita L, Metenou S, Fogako J, Megnekou R, Taylor D W. Changes in the levels of chemokines and cytokines in the placentas of women with *Plasmodium falciparum m*alaria. J Inf Dis **2003** 188:1074-82.

The World health report (2002)Reducing risks, promoting healthy life. WHO, Geneva

Theonest K. Mutabingwa, Melissa C. Bolla, Jin-Long Li, Gonzalo J. Domingo, Xiaohong Li, Michal Fried, Patrick E. Duffy; Maternal Malaria and Gravidity Interact to Modify Infant Susceptibility to Malaria. **2005**; PLOS, 12: 001-009

WHO, Physical status: The use of and interpretation of anthropometry. ed. W.H. Organization. **1995**, Geneva.

WHO. *Complementary feeding of young children in developing countries: a review of current scientific knowledge*. WHO, **1998**, Geneva.

Wilson JN., Rockett K., Jallow M., Pinder M., Sisay-Joof F., Newport M., Newton J., Kwiatkowski D. Analysis of IL10 haplotypic associations with severe malaria. Genes. Immun. **2005**. 6 : 462-466

Xi G, Leke RGF, Thuita LW, Zhou A, Leke RJI, Mbu R, Taylor DW. Congenital exposure to *Plasmodium falciparum* antigens : prevalence and antigenic specificity of in utero-produced antimalarial immunoglobulin M antibodies. Infect Immun **2003**, 71:1242-6.

# C- justification des moyens

###### Partenaire 1 (IRD ; UR010) : suivi de la cohorte, gestion : justification des moyens

- Equipement de plus de 4000 euros = 1 analyseur d’hématologie = **7000 euros**
- Equipement pour le travail de terrain = **59476, soit :**
  - équipement des maternités et du laboratoire de terrain (congélateurs, table d’examen, microscope, médicaments, groupe électrogène …) = **13 705 euros**
  - équipement des enquêteurs (casque, papeterie, stéthoscope …) = **13491 euros**
  - équipement à Cotonou (PC, onduleurs, imprimante, régulateur tension, clés USB pour sauvegarde sur terrain) = **4268 euros**
  - déplacement (carburant et entretien d’un véhicule 4x4 et de motos) = **21 000 euros**
  - réunions du groupe = **7012 euros**
- Fonctionnement pour le suivi en parasitologie, clinique et immunologie (lames, prélèvements, coloration, alicotage, hémoglobine, …) = **60695, soit**
  - parasitologie et clinique = **41039 euros**
  - immunologie = **19 656 euros**
- Fonctionnement pour la génétique : 1500 individus (enfants et parents)= **92700, soit**
  - Extraction de l’ADN = **7500 euros**
  - Séquençage et identification des polymorphismes de l’ADN = **9600 euros**
  - Génotypage (10 gènes candidats et 5 SNP par gène) = **75 600 euros**
- Volet géographie de la santé
  - Ordinateur, logiciels spécifiques, données satellites, billets avion : **8495 euros**
- Frais de personnel (6 infirmiers, 1 ingénieur labo pour Cotonou, 1 technicien labo pour le terrain et 1 chauffeur) : salaire basés sur place donc pas de frais de mission sauf chauffeur = **95113, soit**
  - Prime du personnel local hors projet (sage-femmes, technicien et aide-soignantes …) : pour le total = **6097 euros**
  - Infirmier : (environ 305 euros par mois) pour 30 mois : **54 881 euros**
  - Ingénieur par mois (environ 381 euros par mois) : **13720 euros**
  - Technicien par mois (environ 305 euros par mois) : **10976 euros**
  - Chauffeur par mois (environ 198 euros par mois) : **7134 euros**
  - Chauffeur, frais de déplacement : **2286 euros**
  - Vacations programme géographie : **18160 euros** (dont 700 de frais de mission)

Total partenaire 1 = 341 639 Euros

##### Partenaire 2 (IRD ; UR016) : Enquêtes entomologiques, justification de moyens

- Equipement pour le travail de terrain : 2 loupes binoculaires, petit consommable, papeterie… : **10 000 euros**
- Frais de personnel permanent (technicien + chauffeur) : **9055 euros**
- Frais de personnel temporaire (équipe de 8 captureurs de moustiques) : **6585 euros**
- Frais de mission pendant les missions de captures : **15 000 euros**
- Déplacements (carburant, entretien …) : **5515 euros**
- Consommable de laboratoire (ELISA pour anticorps anti sporozoïtes) : **13 500 euros**

Total partenaire 2 = 59 655 Euros

Les frais de mission du volet entomologique représentent une part élevée des dépenses (25% environ). Ceci s’explique par la nécessité de réaliser des captures par 8 captureurs, 4 nuits consécutives, dans les 15 villages, chaque 6 semaines durant l’ensemble du suivi.

##### Partenaire 3 (IRD ; UR106) : nutrition, justification de moyens

- Equipement pour les maternités : pèse bébé mécanique (SECA), toises, pèse personnes (SECA Bodymaster) …destinés aux mesures à la naissance : **7920 euros**
- Equipement des enquêteurs pour les mesures à domicile : pèse bébé mécanique (SECA), toises, pèse personnes (SECA Bodymaster) …:**7920 euros**
- Frais de mission (missions de formation des personnels pour l’ensemble du programme et pas uniquement sur le volet nutrition), missions de supervision mensuelles …) : **6795 euros**
- Fonctionnement (carburant, consommable, entretien, vacations …) : **4500 euros**
- Voyages du responsable sur le terrain depuis la France (formation et lancement des enquêtes ; évaluations) : **1700 euros**

Total partenaire 3 = 28835 Euros

Les frais de mission du volet nutritionnels (25% du programme environ) inclus les déplacements du responsable du programme qui sera basé en France.

**Partenaire 4 (INSERM; U535) : méthodologie statistique en épidémiologie génétique, justification de moyens**

- Equipement : (1 ordinateur NEC bi-processoret licences STATA et MAPLE version 10) : **5 500 euros**
- Vacations (500 heures pour un ingénieur informaticien (niveau IR) : conception et écriture des programmes d'analyse génétique spécifiques = 12.04 euros par heure soit 6020 Euros + 2486.26 Euros de charges patronales (=41.3% des 6020 Euros) donc au total : **8507 euros**.

Total partenaire 4 = 14007 Euros

**Partenaire 5 (INSERM; U780) : méthodologie statistique en épidémiologie, justification de moyens**

- 2 missions d’une semaine au Bénin : forfait 1500€ par mission (voyage + per diem) : **3000**€

- 1 ordinateur + clé USB 1Go + 2 licences SAS : **1500**€ + **100** Euros + **600** Euros

- documentation : **2000**€

- vacations pour un statisticien : **4000** €

Total partenaire 5 = 11200 Euros

##### Partenaire 6 (UMR CNRS 8071) : méthodologie statistique , justification de moyens

- Vacation statisticien : **5000 euros**
- 6 missions au Benin (1500 E chacune) : **6000 euros**
- 2 ordinateurs + licences + clés  : **4000 euros**

Total partenaire 6 = 15 000Euros
