# Supplementary material for: Associations between an IgG3 polymorphism in the binding domain for FcRn, transplacental transfer of malaria-specific IgG3, and protection against Plasmodium falciparum malaria during infancy: A birth cohort study in Benin
Source: PLoS Med. 2017 Oct 9;14(10):e1002403. doi: 10.1371/journal.pmed.1002403 (PMC5633139; doi:10.1371/journal.pmed.1002403)
Supplement: S2 Protocol — (DOCX) [file pmed.1002403.s004.docx]

## S2 Protocol. First malaria infections in a cohort of infants in Benin: biological, environmental and genetic determinants.

## Overview

Malaria is one of the leading causes of death in developing countries. But the circumstances surrounding early infection are poorly understood. One of the most important factors in the complex malaria pathogenic system – which encompasses the host, the vector and the parasite – lies in exposure to parasites. Diversity in young children’s living conditions is directly related to differences in entomological transmission levels. It is thus of the utmost importance to measure the transmission levels affecting areas where newborns live so that we may understand the dynamics of the clinical expression of early infections. In particular, the comparison of urban and rural areas in the increasingly urbanised African continent should lead to a better knowledge of the major differences between these two types of areas.

Furthermore, host-specific individual factors also play an important part. For instance, placental infection at birth (at similar levels of exposure) is strongly related to the probability of being infected in the first year of life. The biological mechanisms responsible for this are yet to be determined and particularly the hypothesis that *in utero* exposure to parasite antigens leads to a form of immunological tolerance modifying the susceptibility of the child to infection and/or disease. Other factors come into play at this level too, such as nutritional status and contact with other pathogens during childhood.

Finally, the genetic make-up of the host is a crucial factor, especially genetic polymorphism related to immune control. This is a complex form of genetic control (genetic heterogeneity) which interacts with individual (age and duration of exposure), environmental and behavioural factors.

All this is a good illustration of the complexity behind the occurrence of initial malaria parasitaemia. The entire spectrum of factors – exposure to infection, maturation of the immune system and genetic factors – must be taken into account simultaneously during a follow-up of individuals at risk.

In this proposal, we wish to study a cohort of 550 newborns from birth to age 18 months in an area of intense transmission (southern Benin) comprising a rural and an urban area. During the study, parasitological and clinical data will be collected. These will allow us to describe early infections and febrile episodes in newborn children. We will also measure individual (co-infections, nutritional status, immunological and genetic parameters) as well as environmental factors focusing on entomological risk, which will be entered into a geographic information system.

We will identify the main factors for malaria parasitaemia which we have grouped into three categories: (1) ecological (vectorborne transmission, rural/urban habitat), (2) biological (the immune response) and (3) genetic (individual control of susceptibility to infection). This information will allow us to better understand the dynamics behind the initial occurrence and expression of these infections and will enable us to suggest public health interventions to protect groups at risk, as well as for avoiding situations liable to pose threats.

This programme brings together three teams from IRD (specialised in the epidemiology of malaria and genetic epidemiology, entomology and nutrition) which are present in the field, two teams from INSERM and one team from CNRS with recognised achievements in the field of data analysis (survival models and hierarchical models as well as genetic epidemiology). The entire research will be led in conjunction with the Beninese scientific institutions.

## Original protocol

### Objectives

They are three levels: descriptive, etiological, and interventional:

- Identification of the main risk factors for the occurrence of malarial parasitaemias in the first 18 months of life:

- Ecological (vector transmission, urban / rural habitat)
- Biological (constitution of the immune response)
- Genetics (control of individual susceptibility to infection)

- Understanding, in a global approach, the dynamics of appearance and expression of these infections

- Proposed interventions in public health to identify populations in areas at risk and to protect priority groups at risk or to avoid the creation of risk situations

### Data Analysis

#### 2-1 Study of Asymptomatic Parasitic Density Level Risk Factors

A mixed Gaussian linear model will be estimated to study the associations between repeated parasitic densities and co-variables. This model allows to take into account the dependence between the observations, dependence created by the repetition of the measurements in the same child. From the model, predictions of mean parasite density for each subject can be calculated using empirical Bayesian estimators. These predictions will be used as quantitative phenotype in the association analysis.

#### 2-2 Genetic epidemiology analysis: association study

The preceding epidemiological analyzes will make it possible to define, for each subject, the following phenotypes of interest: delay of the appearance of the first parasitaemia and the first malarial attack; Level of asymptomatic parasite density. These phenotypes will be an accurate measure of the host's response to first malaria infections during the first 18 months of life.

The identification of possible associations between phenotypes of interest and candidate gene polymorphisms will be only a first step in this project. Indeed, further developments provide for studies of the expression of the identified polymorphisms. This application does not apply to these program extensions.

The results of the association analysis will be compared to the results of the antibody responses obtained for the child, in order to understand the role of the polymorphism of the cytokine genes (see below) on the process of maturation of the immune system.

##### Exploration of candidate genes

Prior to genetic epidemiology studies, all molecular genetic analyzes (DNA extraction, sequencing of candidate genes for the identification of polymorphisms of interest, genotyping of these polymorphisms) will be carried out in Cotonou and Paris.

The candidate genes will be determined on the basis of the results of previous studies carried out by the group in Cameroon and Senegal, as well as on the basis of the literature.

Family analysis of segregation revealed the existence of a complex genetic control of the response to malaria infection, whether it is the level of parasite density (Garcia et al., 1998a, Rihet et al., 1998a) or IgG responses and IgG subclasses (Stirnadel et al., 2000). Several chromosomal regions, containing many cytokine genes or cytokine receptors, have been predicted for their role in this control. In Cameroon, the team responsible for the project highlighted the interest of the q31-q33 region of human chromosome 5. This region contains a cluster of genes involved in controlling the immune response to various pathogens such as the gene encoding Interleukin (IL) -3, IL-4, IL-5, IL-13, the Colony Stimulator Factor-2 (CSF-2) and the

##### Study of the risk factors for the first asymptomatic and symptomatic parasitaemias

In a first step, a model of semi-parametric survival will be estimated to study the association between the occurrence of the first parasitaemia or the first malarial access and the presence of an infected placenta (Cox, 1972). This model will also identify potential risk factors from the co-variables collected at entry and during follow-up.

In a second step, the sequence of recurrent events in the same individual will be taken into account, which has not been done in previous studies (Le Hesran et al., 1997, Mutabingwa et al., 2005). In this context, the first parasitaemia (or first malaria access) and subsequent episodes of the same individual will be analyzed using appropriate methods based on an extension of the Cox model (Kelly and Lim, 2000). This analysis will make it possible to assess whether the associations identified for the first episode, are also for the following episodes.
