# Supplementary material for: Associations between an IgG3 polymorphism in the binding domain for FcRn, transplacental transfer of malaria-specific IgG3, and protection against Plasmodium falciparum malaria during infancy: A birth cohort study in Benin
Source: PLoS Med. 2017 Oct 9;14(10):e1002403. doi: 10.1371/journal.pmed.1002403 (PMC5633139; doi:10.1371/journal.pmed.1002403)

## S3 Protocol. Modification from the original protocol

### Objectives

The current project is part of the objectives of the original protocol:

- Identification of the main risk factors for the occurrence of malarial parasitaemias in the first 18 months of life:

In this work, we focused on the first 12 months of age. The analysis was first extended to 18 months of age. We observed the same results with less strong associations. Because of the absence of the infant DNA and therefore infant IgG3 polymorphism information, we decided to focus only on the first 12 months of life, period of time when infant could still have maternal IgG in peripheral blood.

- Ecological (vector transmission, urban / rural habitat)

In this work, we used an index based on the ecological measurement performed on this program that represents the malaria individual exposure

- Biological (constitution of the immune response)

In this work, we used the concentrations of total IgG, malaria specific IgG1 and malaria specific IgG3 measured both in infant and in their mothers.

- Genetics (control of individual susceptibility to infection)

In this work, maternal IgG3 gene was the gene of interest. This gene was not one the cited candidate gene. Nevertheless, this gene was part of the protocol as “The candidate genes will be determined on the basis of the results of previous studies carried out by the group in Cameroon and Senegal, as well as on the basis of the literature”.

### Data Analysis

In the original protocol, Cox model was cited to analyse the association between variables of interest and first symptomatic of asymptomatic infection. We followed the protocol.

In a second step, the original protocol had proposed to analyse first malaria parasitemia and subsequent infections using appropriate methods based on an extension of the Cox model. In this work we used a Poisson model which is not part of the cited models in Kelly et al., 2000. Our decision was based on the huge number of non-malaria visit due to the accurate surveillance (both active and passive) observed in this study. To justify our choice, the histogram and the frequency of the number of visit (variable “nb_inf”) with (blue circle) or without (red circle) malaria parasitemia are presented below.


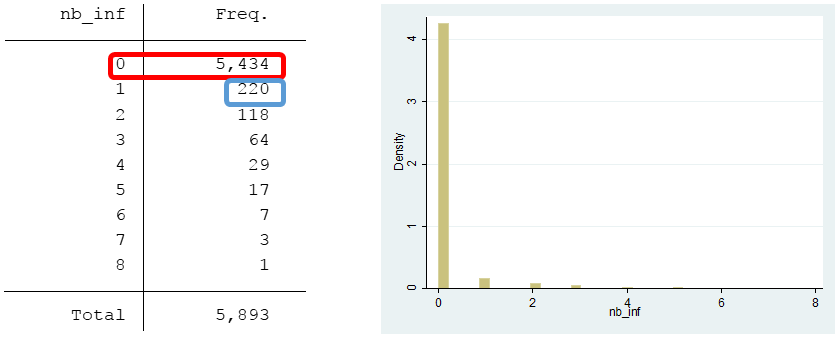

Supplement: S3 Protocol — (DOCX) [file pmed.1002403.s005.docx]
